# Quantification of Macrophage-Driven Inflammation During Myocardial Infarction with ^18^F-LW223, a Novel TSPO Radiotracer with Binding Independent of the rs6971 Human Polymorphism

**DOI:** 10.2967/jnumed.120.243600

**Published:** 2021-04

**Authors:** Mark G. MacAskill, Agne Stadulyte, Lewis Williams, Timaeus E.F. Morgan, Nikki L. Sloan, Carlos J. Alcaide-Corral, Tashfeen Walton, Catriona Wimberley, Chris-Anne McKenzie, Nick Spath, William Mungall, Ralph BouHaidar, Marc R. Dweck, Gillian A. Gray, David E. Newby, Christophe Lucatelli, Andrew Sutherland, Sally L. Pimlott, Adriana A.S. Tavares

**Affiliations:** 1 University/BHF Centre for Cardiovascular Science, University of Edinburgh, Edinburgh, United Kingdom; 2 Edinburgh Imaging, University of Edinburgh, Edinburgh, United Kingdom; 3 School of Chemistry, WestCHEM, University of Glasgow, Glasgow, United Kingdom; 4 Centre for Clinical Brain Sciences, University of Edinburgh, Edinburgh, United Kingdom; 5 MRC Edinburgh Brain Tissue Bank, University of Edinburgh, Edinburgh, United Kingdom; 6 Bioresearch and Veterinary Services, University of Edinburgh, Edinburgh, United Kingdom; 7 Forensic Pathology, University of Edinburgh, Edinburgh, United Kingdom; 8 School of Medicine, University of Glasgow, Glasgow, United Kingdom; and; 9 NHS Greater Glasgow and Clyde, Glasgow, United Kingdom

**Keywords:** TSPO, PET, macrophage, inflammation, myocardial infarction

## Abstract

Myocardial infarction (MI) is one of the leading causes of death worldwide, and inflammation is central to tissue response and patient outcomes. The 18-kDa translocator protein (TSPO) has been used in PET as an inflammatory biomarker. The aims of this study were to screen novel, fluorinated, TSPO radiotracers for susceptibility to the rs6971 genetic polymorphism using in vitro competition binding assays in human brain and heart; assess whether the in vivo characteristics of our lead radiotracer, ^18^F-LW223, are suitable for clinical translation; and validate whether ^18^F-LW223 can detect macrophage-driven inflammation in a rat MI model. **Methods:** Fifty-one human brain and 29 human heart tissue samples were screened for the rs6971 polymorphism. Competition binding assays were conducted with ^3^H-PK11195 and the following ligands: PK11195, PBR28, and our novel compounds (AB5186 and LW223). Naïve rats and mice were used for in vivo PET kinetic studies, radiometabolite studies, and dosimetry experiments. Rats underwent permanent coronary artery ligation and were scanned using PET/CT with an invasive input function at 7 d after MI. For quantification of PET signal in the hypoperfused myocardium, *K*_1_ (rate constant for transfer from arterial plasma to tissues) was used as a surrogate marker of perfusion to correct the binding potential for impaired radiotracer transfer from plasma to tissue (BP_TC_). **Results:** LW223 binding to TSPO was not susceptible to the rs6971 genetic polymorphism in human brain and heart samples. In rodents, ^18^F-LW223 displayed a specific uptake consistent with TSPO expression, a slow metabolism in blood (69% of parent at 120 min), a high plasma free fraction of 38.5%, and a suitable dosimetry profile (effective dose of 20.5–24.5 μSv/MBq). ^18^F-LW223 BP_TC_ was significantly higher in the MI cohort within the infarct territory of the anterior wall relative to the anterior wall of naïve animals (32.7 ± 5.0 vs. 10.0 ± 2.4 cm^3^/mL/min, *P* ≤ 0.001). Ex vivo immunofluorescent staining for TSPO and CD68 (macrophage marker) resulted in the same pattern seen with in vivo BP_TC_ analysis. **Conclusion:** ^18^F-LW223 is not susceptible to the rs6971 genetic polymorphism in in vitro assays, has favorable in vivo characteristics, and is able to accurately map macrophage-driven inflammation after MI.

## Visual Abstract

**Figure:**
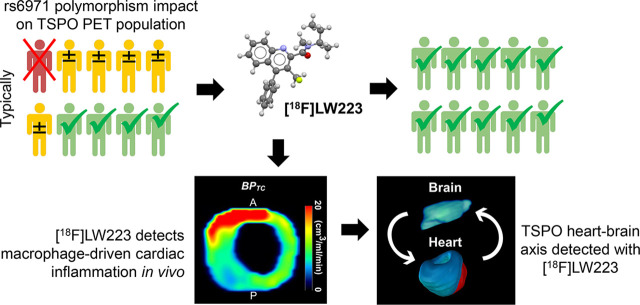

Cardiovascular disease is the leading cause of morbidity and mortality worldwide (1). A large proportion of these fatalities is due to myocardial infarction (MI). Acute inflammation is a key driver of pathology determining disease perturbation after tissue infarction (2). Therefore, an urgent need exists for a noninvasive imaging technology to act as a prognostic tool and predict subsequent patient outcomes.

The 18-kDa translocator protein (TSPO) is expressed within the outer membrane of the mitochondria (3), where it is a key factor in controlling the transport of cholesterol necessary for steroid hormone synthesis. TSPO is highly expressed within inflammatory cells such as macrophages in the periphery (4) and microglia in the brain (5) and has consequently been used as a marker of inflammation in pathologies throughout the body (4,6–10).

Although TSPO is one of the most widely explored targets in the field of PET, clinical adoption of this tissue biomarker has been globally hindered by radiotracers with suboptimal properties. For example, the prototypical TSPO ligand ^11^C-PK11195 (11) has relatively high nonspecific binding and a short half-life (12). Efforts to surpass the limitations of ^11^C-PK11195 have been hampered primarily by the differential binding of second-generation TSPO radiotracers now known to be caused by the rs6971 genetic polymorphism (13). Moreover, the use of TSPO PET radiotracers in the context of cardiovascular disease, in particular after MI, has faced limited adoption due to lack of validated paradigms for quantification of regional tissue inflammation in hypoperfused areas using a single technique or scan. Previously, quantification of regional tissue inflammation after MI has relied on the use of TSPO PET static imaging and SPECT perfusion static scans for correction of the TSPO PET data (6). Consequently, there is a need to develop improved TSPO PET ligands and methodology to boost adoption of this technology for noninvasive imaging of inflammation in cardiology.

This study aimed to screen novel, fluorinated, TSPO radiotracers for susceptibility to the rs6971 genetic polymorphism using the gold standard in vitro competition binding assays in human brain and heart; assess whether the in vivo characteristics of our lead radiotracer is suitable for clinical translation; and validate whether our novel TSPO radiotracer can detect macrophage-driven inflammation in a rat MI model.

## MATERIALS AND METHODS

### Radiotracer Preparation

A 4-step synthetic strategy was developed for the preparation of the chloride precursor and LW223 (Supplemental Fig. 1; supplemental materials are available at http://jnm.snmjournals.org). Initially, the starting material, 3-methyl-4-phenylquinoline-2-carboxylic acid, was prepared as previously reported by us (14). This was converted to the (*R*)-*sec*-butylamide by coupling with (*R*)-*sec*-butylamine using the coupling agent hexafluorophosphate benzotriazole tetramethyl uronium. Under standard basic conditions, the amide was subjected to an *N*-methylation. The key step then involved a radical-mediated bromination of the 3-methyl substituent using *N*-bromosuccinimide and the radical initiator benzoyl peroxide. The resulting bromide intermediate was then converted to the chloride or LW223 using lithium chloride or sodium fluoride, respectively. All intermediates and final compounds were purified by column chromatography and characterized using a combination of nuclear magnetic resonance spectroscopy and mass spectrometry (organic chemistry section within supplemental materials (14,15)). The 2 amide rotamers of LW223 were separated and characterized by liquid chromatography–mass spectrometry (Supplemental Fig. 2).

**FIGURE 1.**
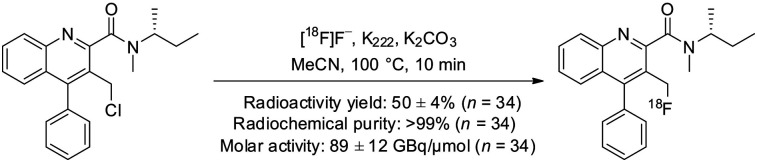
Radiosynthesis of ^18^F-LW223. Data are shown as mean ± SEM.

^18^F-LW223 was prepared as shown in Figure 1, using the GE Healthcare TRACERlab FX_FN_ synthesizer. The radiotracer was purified by semipreparative high-performance liquid chromatography using the following conditions: C18 Synergi Hydro-RP 80 Å, 150 × 10 mm, 4-μm column (Phenomenex), acetonitrile/water (70:30 v/v), and flow rate of 3 mL/min. ^18^F-LW223 was formulated in 10% ethanol in saline. The average radioactivity yield was 50% ± 4% (starting from 22 ± 3 GBq of ^18^F-fluoride, *n* = 34) after a total synthesis time of 55 min. The identity of ^18^F-LW223, radiochemical purity (>99%), and molar activity (89 ± 12 GBq/μmol, *n* = 34) were determined by high-performance liquid chromatography analysis at the end of synthesis.

### In Vitro Competition and Saturation Binding Assays with Human Tissue

All studies using human tissue were conducted in accordance with the National Health Service Research Ethics Committee South East Scotland (Edinburgh Brain and Tissue Bank, 16/ES/0084). Supplemental Table 1 contains the Brain Bank Network reference number for all tissues used from the Edinburgh Brain and Tissue Bank. Fifty-one brain (78% male, aged 53.9 ± 9.5) and 29 heart (83% male, aged 48.2 ± 13.2) samples were obtained and screened for the rs6971 genetic polymorphism and grouped into high-affinity binders (HABs), mixed-affinity binders (MABs), or low-affinity binders (LABs) as previously described (13). Samples were homogenized in ×10 w/v buffer (50 mM Tris-Base, pH 7.4, 4°C) before centrifugation (32,000*g,* 10 min, 4°C). Tissue pellets were then resuspended in ×10 w/v buffer and centrifuged again before resuspending in 2 mL of buffer. Samples were assessed for protein concentration using the Bio-Rad protein assay, aliquoted, and stored at −80°C until use. The competition binding assay protocol was adjusted from a previously reported protocol (16), as detailed within the supplemental materials.

### Animals and Surgical Procedures

All experiments were authorized by the local University of Edinburgh Animal Welfare and Ethical Review Committee and in accordance with the Home Office Animals (Scientific Procedures) Act of 1986. Thirty-three adult male Sprague–Dawley rats (312.1 ± 15.0 g), and 13 C57BL/6 rats (8 male, 5 female; 25.5 ± 1.5 g), were used for this study. The animals were housed under standard conditions of 12 h of light and 12 h of darkness, with food and water available ad libitum. On the day of the experiment, anesthesia was induced and maintained with 1.5%–2.5% isoflurane (50:50 oxygen/nitrous oxide, 1 L/min). For imaging experiments, an intravenous line was established in the femoral vein or tail vein for injection of the radiotracer, and the femoral artery was cannulated to allow automated blood sample collection, as previously described (17). In a separate set of experiments (radiometabolite studies), the femoral artery was cannulated for blood sampling and the radiotracer was administered via the tail vein. Surgical cannulation of the femoral vein and artery was performed as detailed in the supplemental materials.

Body temperature was maintained by a heated scanner bed or heated mat and monitored by a rectal thermometer. Vital signs, including heart rate and respiration rate, were monitored continuously during the experiments.

For MI studies, anesthesia was induced and maintained using isoflurane (0.5%–3% in oxygen, 1 L/min) before buprenorphine (0.05 mg/kg; Alstoe Ltd.) was administered preoperatively for analgesia. Tracheal intubation was achieved under direct vision, and ventilation was maintained with a rodent ventilator (model 683 [Harvard Apparatus]; tidal volume, 2.5 cm^3^; respiratory rate, 60/min). MI was induced as we have previously described (18), and further details can be found in the supplemental materials. Animals were imaged within 1 d of day 7 after MI, referred to as day 7 throughout the article.

### In Vivo PET Imaging

#### Study Design

Thirty-two PET scans were performed with ^18^F-LW223 (17.8 ± 1.5 MBq, intravenous bolus, mean ± SEM). Naïve and MI rat scans were acquired immediately after intravenous bolus injection of ^18^F-LW223. Blocking studies were performed on rats with PK11195 (1 mg/kg, intravenously) 30 min before they received ^18^F-LW223. For displacement studies, rats received ^18^F-LW223 and were scanned continuously for 120 min, with a single dose of PK11195 (1 mg/kg, intravenously) administered at the 60-min time point. Dosimetry studies were conducted using mice and 240-min PET scans.

#### Arterial Input Functions

A commercially available system (Twilite2; Swisstrace) was used for the measurement of blood radioactivity as previously described (17). The whole-blood arterial input function measured by the automatic blood sampler was corrected for the plasma–to–whole-blood ratio and for metabolism in vivo.

#### Image Acquisition and Reconstruction

Data were acquired using a PET/CT small-animal scanner (nanoPET/CT; Mediso). A CT scan (semicircular full trajectory, maximum field of view, 480 projections, 50 kVp, 300 ms, and 1:4 binning) was acquired for attenuation correction. Immediately after radiotracer administration, a 120- or 240-min emission scan was obtained using 3-dimensional 1:5 mode. For rat studies, rebinning was 18 × 10 s, 2 × 30 s, 1 × 60 s, 2 × 2 min, 10 × 5 min, 6 × 10 min, or 18 × 10 min. For mouse studies, rebinning was 4 × 5 min, 4 × 10 min, or 9 × 20 min. Rat PET studies were reconstructed using the iterative Tera-Tomo 3-dimensional reconstruction algorithm (Mediso), which includes point-spread correction, and the following settings: 4 iterations, 6 subsets, full detector model, low regularization, spike filter on, voxel size of 0.4 mm, and 400- to 600-keV energy window. Mouse dosimetry PET data were reconstructed using filtered backprojection, a voxel size of 0.4 mm, and a 400- to 600-keV energy window. All PET data were corrected for randoms, scatter, and attenuation.

#### Image Processing and Data Analysis

Reconstructed scans were imported into PMOD, version 3.8 (PMOD Technologies). Volumes of interest (VOIs) were manually drawn around the brain, heart, and lung using CT images. To sample the infarct area (or an equivalent area in naïve hearts), averaged PET images (0–120 min) were used to place 3 spheric VOIs at the center of the infarct territory within the ventricular wall (1.5 mm^3^). Only the right lung was used for analysis in MI rats to avoid surgically induced trauma (the left lung is deflated during the surgical procedure). Time–activity curves were generated, and SUVs were calculated as concentration in the VOI divided by injected dose divided by animal weight. Kinetic modeling was performed using the 2-tissue-compartment model to estimate the kinetic rate constants *K*_1_–*k*_*4*_ and the total volume of distribution (*V*_*T*_) in different tissues. Blood volume fraction (*V*_*B*_) was not modeled in this study. Two-tissue-compartment modeling was selected as the preferred model on the basis of a comparison of goodness and robustness of fitting versus several other models (Supplemental Fig. 3; Supplemental Table 2). The binding potential relative to nondisplaceable volume (BP_ND_) was defined as *k*_3_/*k*_4_ (19). The transfer-corrected BP_ND_, termed BP_TC_, was formulated as follows:

Eq. 1 $${\text{BP}}_{\text{TC}}=\frac{k_{3}}{k_{4}}÷K_{1},$$

where *k*_3_ is the radiotracer association rate with specific binding, *k*_*4*_ is the dissociation rate constant of target ligand, and *K*_1_ is the rate constant for transfer from arterial plasma to tissues. When tissue blood flow is impaired, such as after MI, *K*_1_→blood flow, as per the Renkin–Crone equation:

Eq. 2 $$K_{1}=F\times E=F\;\left(1-e^{-\;\frac{PS}{F}}\right),$$

where *F* is flow, *E* is extraction, and *PS* is permeability surface area.

#### Dosimetry

Reconstructed whole-body PET scans were imported into PMOD, version 3.8, and VOIs were drawn around organs that displayed a radioactivity concentration higher than the background concentration, that is, source organs. The following organs were identified as source organs: brain, heart, lung, gallbladder, liver, gut, adrenals, kidneys, and bladder. A whole-body VOI was drawn around the animal body and was used to quantify whole-body remainder activity as whole-body activity minus source-organ activity. At each time point, the measured activity of the source organs was expressed as the percentage injected dose. The residence time (τ), defined as the ratio of accumulated activity in the target organ (Ā) and injected activity (A_0_) (i.e., τ = Ā/A_0_ (20)), was calculated as previously described (21). Calculated τ was normalized on the basis of the factors shown in Supplemental Table 3 (22–26) and entered into OLINDA/EXM software, version 1.0, to estimate organ doses and effective doses.

### Radiometabolite Blood and Tissue Processing and Analysis

Arterial blood samples were collected at 2, 5, 10, 20, 30, 60, and 120 min after radiotracer injection (69.7 ± 9.1 MBq [mean ± SEM], *n* = 17 rats), and heart, lung, and brain tissue samples were collected at 60 and 120 min. Blood and tissues were processed as detailed in the supplemental materials.

### Ex Vivo Immunofluorescence Imaging

Naïve and MI heart tissue was fixed in 10% neutral buffered formalin for 24 h, wax-processed, and sectioned. The CD68 (a macrophage marker) and TSPO staining was performed using a double tyramide signal amplification visualization as described previously (27). Mouse and rabbit IgG at an equal concentration served as an isotype control. Full details on the immunohistochemistry tissue processing protocols used can be found in the supplemental materials.

Whole tissues were imaged using an Axio Scan.Z1 slide scanner (Zeiss). Quantification was performed using Image J (version 1.49; NIH) by selecting regions of interest and quantifying the mean intensity for each channel of interest.

### General Statistical Analysis

GraphPad Prism, version 6 (GraphPad Software Inc.), was used for data fitting, statistical analysis, and production of graphs. In competition binding assays and saturation assays, outliers within experimental triplicates were removed using a Grubbs test with an α of 0.2. Two-way ANOVA with post hoc Šidák correction, Pearson correlations, and unpaired and paired *t* tests were used in this study for comparison between 2 groups, as indicated within the relevant figure legends, with a *P* value of less than 0.05 considered statistically significant. All error bars represent the SEM, unless otherwise indicated in the figure or table legends. All uses of ± within the text refer to SEM.

## RESULTS

### Binding of Our Lead Compound, LW223, to TSPO Is Not Affected by the rs6971 Polymorphism in In Vitro Assays

**FIGURE 2.**
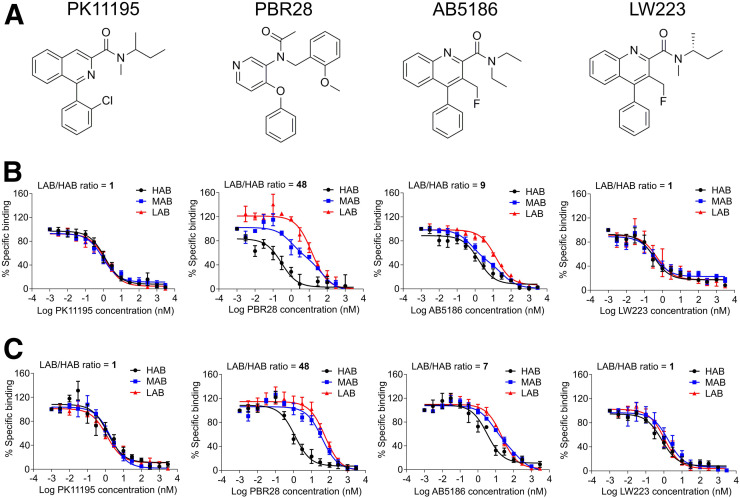
LW223 binding to TSPO is not affected by human rs6971 genetic polymorphism. (A) Chemical structures of established (PK11195, PBR28) and novel (AB5186, LW223) TSPO ligands investigated. (B) Competition binding assays using these TSPO ligands in human brain homogenates genotyped and grouped as HAB, MAB, and LAB (PK11195: HAB *=* 6, MAB = 8, LAB = 4; PBR28: HAB *=* 4, MAB [2-site fitting] = 5, LAB = 4; AB5186: HAB *=* 6, MAB [2-site fitting] = 6, LAB = 5; LW223: HAB *=* 5, MAB = 5, LAB = 4). Only PK11195 and LW223 were not affected by polymorphism, as is also demonstrated in human heart homogenates (C) (PK11195: HAB *=* 4, MAB = 5, LAB = 4; PBR28: HAB *=* 4, MAB = 5, LAB = 4; AB5186: HAB *=* 4, MAB = 5 [2-site fitting], LAB = 4; LW223: HAB *=* 5, MAB = 5, LAB = 4).

The inhibition constant (*K*_*i*_) (28) of PK11195, 2 PK11195 analogs (AB5186 (29) and LW223, our lead compound), and PBR28 (Fig. 2A) were investigated in this study. Saturation assays were used to determine the dissociation constant of PK11195 (Supplemental Fig. 4).

In the human brain, PK11195 *K*_*i*_ was unaffected by the genetic polymorphism, unlike PBR28; AB5186 *K*_*i*_ was affected by the genetic polymorphism, unlike LW223, which was unaffected (Fig. 2). There were no differences between HAB and LAB in PK11195 and LW223 binding studies (Supplemental Figs. 5A and 5G). Conversely, *K*_*i*_ values in HAB and LAB for PBR28 and AB5186 revealed differences between groups (Supplemental Figs. 5C and 5E). LW223 mean *K*_*i*_ was 0.6 nM, twice that of PK11195.

In the human heart, the *K*_*i*_ of PK11195 was independent of the genetic polymorphism; PBR28 and AB5186 were also affected to a similar degree in the heart and in the brain; LW223 binding in the heart was unaffected by the polymorphism (Fig. 2C). *K*_*i*_ values for all heart samples demonstrated no differences between HAB and LAB in PK11195 and LW223 binding studies (Supplemental Figs. 5B and 5H). In the heart, LW223 mean *K*_*i*_ was the same as PK11195 at 1.7 nM. Individually calculated *K*_*i*_ values for PBR28 and AB5186 revealed a difference between HAB and LAB (Supplemental Figs. 5D and 5F).

### ^18^F-LW223 Binds to TSPO In Vivo

**FIGURE 3.**
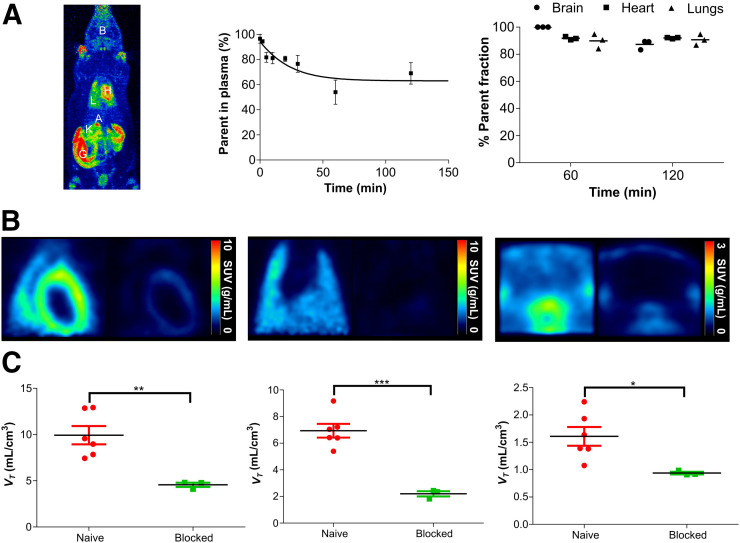
^18^F-LW223, assessed in naïve rats, has favorable metabolic profile and specifically targets TSPO in vivo. (A) Maximum-intensity projection of ^18^F-LW223 PET scan in naïve rat showing major uptake organs (left); metabolism of ^18^F-LW223 within rat blood showing high percentage of parent radiotracer within plasma up to 2 h after injection (middle, *n =* 3 per time point); and percentage of parent radiotracer within different tissues demonstrating low level of contaminating radiometabolites (right, *n =* 3). (B) SUV images of rat heart (left), lung (middle), and brain (right) at baseline (left side of each panel) and under TSPO blockade using prototypical TSPO ligand PK11195 (1 mg/kg, right side of each panel) demonstrating specificity of ^18^F-LW223 for this target. SUV PET images were filtered using gaussian 1 × 1 × 1 mm filter. (C) Total *V*_*T*_ within heart (left), lung (middle), and brain (right) of naïve and TSPO-blocked rats. Results represent mean ± SEM (*n =* 6 for naïve; *n =* 3 for blocked). *P* values were obtained using unpaired *t* test. A = adrenal glands; B = brain; G = gut; H = heart; K = kidney; L = lung. *P < 0.05. **P ≤ 0.01. ***P ≤ 0.001.

In mice and rats, ^18^F-LW223 rapidly distributed to TSPO-expressing tissues, including brain, heart, and lung after administration. It was eliminated via both the urinary and the hepatobiliary excretion routes (Fig. 3A).

Radiometabolism of ^18^F-LW223 in rat arterial blood was slow, with approximately 69% of the parent compound remaining at 120 min after injection; less than 12.7% radiometabolites were measured in the brain, heart, and lung at 60 and 120 min after administration (Fig. 3A; Supplemental Figs. 6 and 7). The measured parent free fraction in plasma was 38.5% ± 7.0% (mean ± SEM, *n* = 5).

Peak SUVs in naïve rat brain, heart, and lung were 0.83 ± 0.18, 2.51 ± 0.31, and 3.98 ± 0.35 g/mL, respectively. After peak uptake, elimination of the radiotracer was faster in the brain and lung than in the heart (Supplemental Fig. 8). Time–activity curves were noisier in the sampled left ventricle (LV) subregion than in whole-organ VOIs (Supplemental Figs. 8D–8F). ^18^F-LW223 kinetics in naïve rats were blockable (Figs. 3B–3C) and reversible (Supplemental Fig. 9) and could be described by the 2-tissue-compartment model (Supplemental Table 4).

In naïve mice, the highest percentage injected dose was in the gut, followed by the liver, lung, kidneys, heart, brain, gallbladder, urinary bladder, and adrenals. Dosimetry estimates demonstrated that the critical organ was the lower large intestine. The whole-body effective dose was estimated to be 20.5 and 24.5 μSv/MBq in male and female animals, respectively (Table 1)

**TABLE 1:** Estimated ^18^F-LW223 Radiation Dose for Humans Is Within Acceptable Limits for Future Clinical Use

	Estimated absorbed dose (×10^−2^ mGy/MBq)	
Target organ	Male (*n =* 8)	Female (*n =* 5)
Adrenals	2.22	3.67
Brain	0.59	0.56
Breasts	0.72	0.86
Gallbladder wall	2.01	2.46
Lower large intestine wall	7.82	9.26
Small intestine	1.11	1.30
Stomach wall	0.94	1.15
Upper large intestine wall	1.00	1.24
Heart wall	2.03	1.89
Kidneys	1.57	1.54
Liver	1.30	1.95
Lung	3.10	3.31
Muscle	0.80	0.98
Ovaries	—	1.66
Pancreas	1.03	1.26
Red marrow	0.82	0.98
Osteogenic cells	1.20	1.52
Skin	0.61	0.74
Spleen	0.91	1.11
Testes	0.77	—
Thymus	0.90	1.07
Thyroid	0.79	0.89
Urinary bladder wall	1.16	1.48
Uterus	—	1.29
Total body	0.89	1.07
Effective dose (mSv/MBq)	0.02	0.02

### ^18^F-LW223 BP_TC_ Allows for Detection of Macrophage-Driven Inflammation in the Hypoperfused Myocardium After MI

**FIGURE 4.**
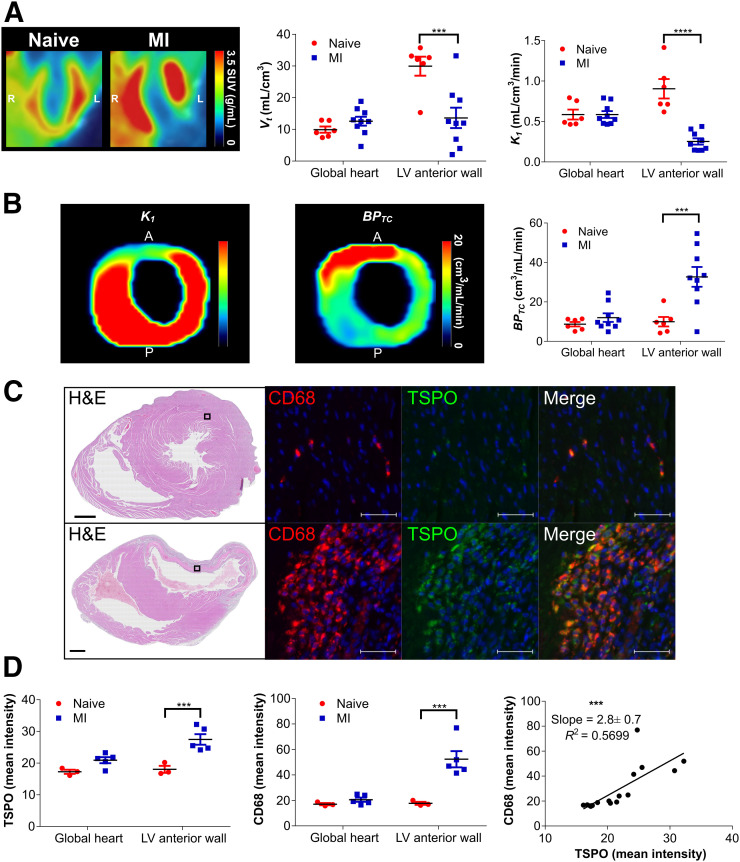
^18^F-LW223 PET with BP_TC_ quantification detects macrophage-driven inflammation within heart 7 d after MI without need for additional perfusion scan. (A) Long-axis representative SUV image of heart in naïve and MI rats showing increased global uptake and lack of signal within LV anterior wall due to MI-mediated reduction in perfusion (left) (images were filtered using gaussian 1 × 1 × 1 mm filter)); non–perfusion-corrected *V*_*T*_ within global heart and LV anterior wall, which was site of infarct (middle); and *K*_1_ acting as surrogate marker of perfusion, being reduced within LV anterior wall (right). (B) Representative *K*_1_ (left) and BP_TC_ images (middle) of LV of MI rat demonstrating true TSPO signal across heart; BP_TC_ values across global heart and LV anterior wall demonstrating that most TSPO is expressed within infarct (right). *n* = 6 for natïve and *n* = 9 for MI. (C) Representative histology examples of hearts from naïve (top) and MI (bottom) rat. Hematoxylin and eosin (H&E) overview (scale bar = 1,000 μm) contains box that indicates position of CD68 examples (macrophage marker) and TSPO examples (scale bar = 50 μm), demonstrating specificity of TSPO for macrophages, which are mostly present within infarct. (D) Quantification of TSPO immunofluorescent stain indicating that most signal is present within LV anterior wall (left), as is also true for CD68 quantification (middle); comparison of TSPO and CD68 indicates significant correlation within heart (right). *n* = 3 for naïve and *n* = 5 for MI. All results represent mean ± SEM. *P* values were obtained using 2-way ANOVA with post hoc Sidak for naïve vs. MI, apart from correlation analysis, which used Pearson correlation. A = anterior; P = posterior. **P ≤ 0.01. ***P ≤ 0.001. ****P ≤ 0.0001.

In non–perfusion-corrected SUV heart images, there was an increase in ^18^F-LW223 signal globally, with a lack of signal within the LV anterior wall (area of the infarct) 7 d after MI; there was a significant decrease in the measured *V*_*T*_ in the LV anterior wall (i.e., infarct area) in the MI group (Fig. 4A). *V*_*T*_ and SUVs at pseudoequilibrium (40–60 min) in naïve and MI animals significantly correlated (Supplemental Fig. 10). However, heart VOIs on their own did not correlate in MI animals, suggesting that *V*_*T*_ and SUV are not equally affected by tissue hypoperfusion induced by the coronary artery ligation. *K*_1_ was also significantly reduced in the LV anterior wall of the MI group (Fig. 4A; Supplemental Table 4). The measured reduction in all modeling constants was not linear, with *k*_*2*_ reducing only 1.4-fold, versus 3.6-fold for *K*_1_ (Supplemental Table 4). The ratio of the constants *k*_3_/*k*_4_ remained unchanged between groups (Supplemental Table 4). Figure 4B contains examples of parametric images of an LV from the MI group, with the perfusion deficit caused by ligation of the left anterior descending artery, as clearly seen in the *K*_1_ image. The BP_TC_ parametric imaging data showed a clear signal mainly within the infarct. Quantification of BP_TC_ between groups demonstrated a significant difference (increase) within the LV anterior wall between naïve and MI rats. There was no statistically significant changes in measured ^18^F-LW223 concentration in whole blood after MI (Supplemental Fig. 11).

BP_TC_ was validated by ex vivo analysis of naïve and MI hearts and immunofluorescent staining of TSPO and CD68 (Fig. 4C). In the naïve and MI hearts, most TSPO staining (green, cytoplasmic) was found in macrophages, which were CD68-positive (red, cytoplasm and cell surface). These markers only occasionally colocalized to the same region of the cell (yellow) because of their subcellular distribution. Quantification of TSPO staining revealed a significant increase in the LV anterior wall of the MI group, which was also the case for CD68; TSPO within the global heart and LV anterior wall significantly correlated with CD68 (Fig. 4D). A trend toward a small increase in TSPO and CD68 staining was observed in the remote myocardium (i.e., posterior LV, Supplemental Fig. 12). ^18^F-LW223 autoradiography in these samples also demonstrated an increase in signal within the MI group (1-way ANOVA, *P* < 0.05), although it lacked the statistical power to determine significant regional differences in post hoc analysis (Supplemental Fig. 13). The localization, and correlation, of TSPO and CD68 within the infarct in ex vivo analysis validates the expression pattern seen for ^18^F-LW223 BP_TC_ in vivo (Fig. 4B) and reflects macrophage-driven inflammation in the heart after MI.

Our simulation work using ideal noise-free conditions shows that the difference between true BP_ND_ and estimated BP_ND_ is more pronounced for low *K*_1_ values (Supplemental Fig. 14; Supplemental Tables 5–8). Low *K*_1_ values simulate conditions of severe hypoperfusion, such as those observed during MI. Furthermore, data show that the reduction of *K*_1_ needs to be much higher in areas of low BP_ND_ (e.g., brain) than in areas of expected high BP_ND_ (e.g., infarct region), in order to impact BP_ND_ measures (Supplemental Fig. 14).

### ^18^F-LW223 PET Corroborates Existence of an Inflammatory Heart–Brain Axis in Rats After MI

**FIGURE 5.**
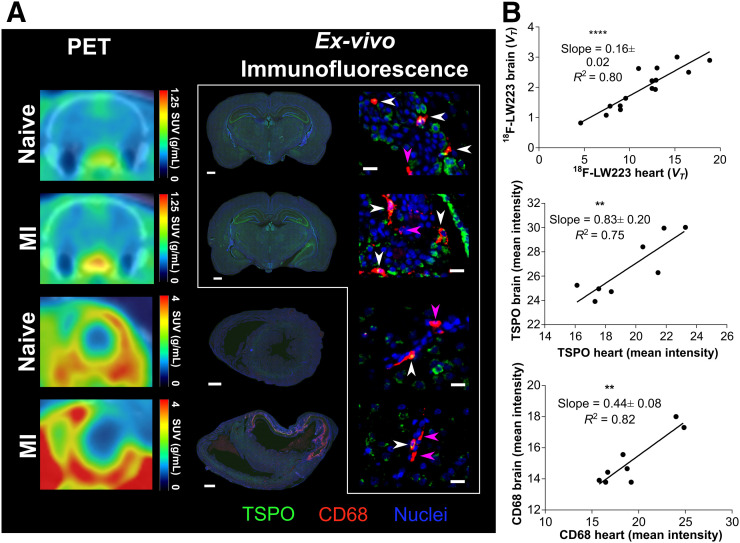
Heart–brain axis in health and after MI. (A) Representative ^18^F-LW223 brain (top, coronal section) and heart (bottom, short axis) PET/CT images of naïve and MI rats (left); CD68 (red) and TSPO (green) immunofluorescence within same animals (middle, scale bar = 1,000 μm); and regional brain immunofluorescence within lateral ventricles of naïve (top) and MI (second from top) rats and within thalamus of naïve (second from bottom) and MI (bottom, scale bar = 20 μm) rats. White arrowheads denote cells that are both CD68- and TSPO-positive; pink arrowheads denote cells positive only for CD68. Cells positive only for TSPO were abundant and are not specifically denoted. (B) Comparison of ^18^F-LW223 *V*_*T*_ in heart and brain of naïve and MI rats showing strong correlation, (*n* = 15), as is also true for TSPO (*n* = 8) and CD68 (*n* = 8). *P* values were obtained using Pearson correlation. **P ≤ 0.01. **P ≤ 0.0001.

In line with previous observations in mice and humans (6), we investigated the existence of a TSPO heart–brain inflammation axis in rats 7 d after MI. There was a significant positive correlation in ^18^F-LW223 signal (in vivo), TSPO (ex vivo immunofluorescence), and CD68 expression (ex vivo immunofluorescence) (Figs. 5A and 5B) between the heart and brain of naïve and MI rats. Ex vivo TSPO analysis revealed a significant increase after MI within the lateral ventricles of the brain but no significant change in the global brain, hippocampus, thalamus, or cortex (Supplemental Fig. 15). Examples of TSPO and CD68 cellular expression with and without colocalization in the brain are shown in Figure 5A.

## DISCUSSION

The first aim of this study was to develop a novel TSPO ligand that was insensitive to the rs6971 genetic polymorphism in vitro using the gold standard human tissue competition binding assays and 2 types of tissue (brain and heart), which in this case was LW223. Three recently developed PET compounds have also been proposed to be insensitive to the human rs6971 genetic polymorphism: ^18^F-FEBMP (small pilot autoradiography study, *n =* 2) (30), (*R*)-^18^F-nebifquinide (binding assessed using human thrombocyte membranes) (31), and (*R,S*)-^18^F-GE387 (binding assessed using human embryonic kidney cell lines overexpressing human TSPO wild-type and TSPO A147T) (32). Unfortunately, ^18^F-FEBMP underwent considerable metabolism when assessed in rats, with less than 10% of the original parent compound found in blood at 30 min after injection (30). In vivo kinetic properties in animal models of disease and dosimetry estimates of both (*R*)-^18^F-nebifquinide and (*R,S*)-^18^F-GE387 are yet to be fully assessed. Meanwhile, our study presents detailed in vivo kinetic modeling, target-engagement quantification, and animal disease model studies with ^18^F-LW223. Thus, to date, ^18^F-LW223 is the most advanced fluorinated TSPO-PET radiotracer with desirable in vivo characteristics and insensitivity to the rs6971 polymorphism in human tissue (as opposed to cell lines and blood cells), as is necessary for clinical translation.

Our robust translational package reported here with ^18^F-LW223 is further complemented by detailed in vivo characterization. ^18^F-LW223 has excellent in vivo properties for imaging TSPO noninvasively. Although direct comparison with other radiotracers is not easy because of differences in species and experimental methods, ^18^F-LW223 had a *V*_*T*_ in the brain of 1.1–2.2 mL/cm^3^, which was higher than reported values for ^11^C-PK11195 (0.6–0.74 mL/cm^3^) (33) and ^18^F-GE180 (0.10–0.28 mL/cm^3^) (34,35). This finding was in line with other promising TSPO radiotracers, such as ^11^C-ER176 (1.6–3.5 mL/cm^3^) and ^18^F-PBR111 (2.22–4.03 mL/cm^3^) (36,37). ^18^F-LW223 brain SUVs in rats (ranging between 0.5 and 1.3 mg/mL over time) also compared favorably with ^11^C-PK11195 (0.6–0.8 mg/mL) (38) in the same species. Comparison of ^18^F-LW223 performance in mapping TSPO in heart and lung is more challenging because of limited data on previously developed TSPO PET radiotracers outside the brain. Still, ^18^F-LW223 SUV_peak_ in the rat heart and lung (2.5 and 4.0, respectively) also compared favorably with ^11^C-PK11195 (SUV_peak_ of 5 in heart and 3 in lung) in humans (39). Importantly, ^18^F-LW223 has a considerably high parent free fraction in plasma (38.5%), compared with ^11^C-PBR28 (21%) (40) in rats and ^11^C-PBR28 (3%–11%) (16), ^11^C-PK11195 (1%) (41), and ^11^C-ER176 (3.3%) (36) in humans. ^18^F-LW223 also has an excellent radiometabolic profile (62% parent at 120 min in plasma), which is higher than that of ^11^C-PK11195 (∼40% (42), in the same lower species). The measured radiometabolites in plasma were less lipophilic than the parent compound, suggesting they would have lower brain penetration. This suggestion is confirmed by the low fraction of radiometabolites measured in brain tissue and other target organs; this low fraction will minimally affect in vivo PET data quantification. ^18^F-LW223 binding to TSPO in vivo was blocked and displaced by PK11195, indicating the specificity of this novel ligand for TSPO. The estimated dosimetry of ^18^F-LW223 (0.020 mSv/MBq) was also well within typical values for ^18^F-labeled radiotracers currently in routine clinical use (e.g., ^18^F-FDG, 0.029 mSv/MBq (43)), therefore easing translation to humans.

An additional aim of this study was to develop a novel multiparametric analysis approach that would allow for accurate quantification of regional TSPO expression within the infarcted myocardium with a single-scan paradigm. We used dynamic imaging and a 2-tissue-compartment model to quantify the different rate kinetics of the ^18^F-LW223 signal. Traditionally, a 2-tissue-compartment model is used to calculate *V*_*T*_ (19). Our data demonstrated that the drop in *K*_1_ was severely perturbed within the infarct region to a far greater extent than the other constants, particularly *k*_*2*_. Because of the imbalanced decrease in movement of radiotracer from the blood into the tissue (*K*_1_) and the reverse (*k*_*2*_), and the fact that during these hypoperfused conditions *K*_1_→*F*, *V*_*T*_ was not able to reflect disease activity accurately. For our high-affinity ligand, ^18^F-LW223, and in the highly hypoperfused and inflamed myocardium, the result is a violation of the Michaelis–Menten free ligand approximation principle. In these circumstances, the Morrison kinetics of tightly binding reversible ligands apply (44,45).

Our reported *K*_1_ values (Supplemental Table 4) were relatively high in the heart and lung but not in the brain, thus indicating that the respective permeability is also high for ^18^F-LW223 and thus supports the *K*_1_→*F* assumption. Moreover, our in vivo modeling data are also corroborated by ex vivo tissue analysis with PET-independent measures of antibody-tagged TSPO immunofluorescence. ^18^F-LW223 can be classified as a moderately extracted radiotracer if we use ^15^O-water as a benchmark for high extraction. Therefore, theoretically, deviation of apparent *K*_1_ (i.e., flow × extraction) from the flow line of identity would be more pronounced for high flows (e.g., healthy myocardium) than for low flows (e.g., infarcted region) (46). This behavior is, in fact, expected and similar to that of routinely used SPECT or PET myocardial perfusion agents (e.g., ^99m^Tc-sestamibi or ^18^F-flurpiridaz) (47).

Previously, this hemodynamic change after MI has been corrected using an additional SPECT scan (6), resulting in an increased cost and patient burden. Here we derived BP_TC_ as a new outcome measure for quantification of the TSPO PET signal using a single-scan protocol. Currently, we make use of the gold standard invasive arterial sampling for data quantification, although this is not always practical in a clinical setting. With the validation of BP_TC_ using ex vivo histology, clinical translation can now take place using a simplified noninvasive image-derived input function to derive robust outcome measures in line with our previously reported findings (48).

Our simulation work using ideal noise-free conditions shows that the reduction of *K*_1_ needs to be much higher in areas of low BP_ND_ (a proxy for TSPO brain uptake) than in areas of expected high BP_ND_ (a proxy for TSPO heart uptake) to impact BP_ND_ measures. Sander et al. have recently reported that large *K*_1_ and small *k*_*2*_ values (i.e., large volume of distribution, *V*_*D*_) are most sensitive to changes in blood flow (49). This report is in line with our data and points out important differences when assessing radiotracer binding in the heart (where *V*_*D*_ is high) versus the brain (where *V*_*D*_ is low). Therefore, even though minor BP_ND_ changes in the brain were reported by Sander et al. because of cerebral blood flow increases of up to 100% at mid scan, these observations might not translate directly to other organs and hypoperfusion scenarios as opposed to hyperperfusion. In particular, studies have shown that after MI, vascular remodeling includes regional vasodilation, vasoconstriction, or pruning. All of these have been shown to impact microvascular conductivity (50), which could contribute to imbalanced *K*_1_-versus-*k*_*2*_ changes beyond flow changes.

Previous studies using chemokine receptor type 4 radiotracers, an alternative inflammatory target, have shown that the highest radiotracer signal was detected in areas of infarct at 3 d after MI without perfusion correction (51,52). Differences between these studies and ours may be due to the difference in target, size of radiotracer molecule, experimental model, and time point assessed. These last 2 points result in considerable differences in blood flow, hemodynamic responses, and tissue remodeling stages, which will impact imaging outcomes.

In the rat MI model used in this study, we demonstrated that most TSPO expression (BP_TC_) was within the infarct region of the MI cohort, as was in agreement with ex vivo analysis. However, in noninfarcted myocardium in other MI or cardiomyopathy models of heart failure, there is significant TSPO expression, indicating that TSPO may have a wider role in the failing heart (53,54).

A limitation is that we assessed only 1 time point in our MI study. Follow-up studies with longitudinal TSPO imaging after MI in rats are being planned and will be necessary to robustly assess the transient nature of inflammation and this molecular target. In addition, it is not clear whether targeting TSPO in macrophages is selective for M1 or M2 phenotypes, with previous evidence being conflicted on this aspect (6,55,56). This issue is something we also plan to address in our follow-up studies, in addition to selectivity of ^18^F-LW223 for different leukocyte subpopulations, as has been previously performed (6). Another limitation of our study is that we have performed radiometabolite analysis of ^18^F-LW223 in only naïve rats. Our data suggest there are no significant differences in radioactive concentration in blood of naïve versus MI animals; however, it is possible that the metabolite profile of ^18^F-LW223 changes during MI, and this change could affect the PET outcome measures.

With the advent of total-body PET clinical scanners (57), assessment of multiorgan responses to disease will become increasingly important in medical research. Such assessments are attractive for TSPO-PET studies, given that TSPO has served as a marker of regional tissue inflammation in pathologies throughout the body (4,6–10). In this study, we observed a significant positive correlation between the brain and heart after MI—a correlation that was detected by ^18^F-LW223 PET imaging in vivo and by TSPO and CD68 staining ex vivo. Additionally, our ex vivo results demonstrated a trend toward increased TSPO expression staining in the whole brain after MI, albeit without reaching significance. The degree of change was small and similar to the change previously reported by Thackeray et al. (6). However, at a regional level there was increased expression of TSPO within the lateral ventricles. It is possible that the increased signal in the brain is due to blood–brain barrier hyperpermeability and dysfunction of the blood–cerebrospinal fluid system, which exist to preserve central nervous system homeostasis (58). This possibility is corroborated by the increase in TSPO expression we observed in the lateral ventricles and agrees with previous studies demonstrating the role of the choroid plexus in the recruitment of macrophages at distant sites (59,60). At a cellular level, although some TSPO expression colocalized with the CD68 marker in the brain, there were cells positive for TSPO that lacked coexpression with CD68. This finding is to be expected, as TSPO is expressed in several brain cell types (61). Overall, our results with ^18^F-LW223 PET in this rat model of MI corroborate the findings by Thackeray et al. in mice and humans, thus echoing the importance of integrative systems biology analysis in PET studies (6). The clinical importance of the heart–brain axis after MI is not yet clear and should be investigated further.

## CONCLUSION

We have developed a novel TSPO radiotracer that is not susceptible to the rs6971 human genetic polymorphism, and we also developed an original multiparametric analysis approach. These tools now warrant further investigation and translation in the fields of cardiology and neurology.

## DISCLOSURE

A patent for TSPO binders has been submitted (application GB1810312.7 and PCT/EP2019/066546). This work was funded by the British Heart Foundation (PG/16/12/32022 and PG/17/83/33370) and the Engineering and Physical Sciences Research Council (EPSRC Impact Acceleration Awards, EP/K5039031 and EP/R511705/1). The British Heart Foundation is greatly acknowledged for providing funding toward establishment of the preclinical PET/CT laboratory at the University of Edinburgh (RE/13/3/30183) and radiometabolite laboratories (RG/16/10/32375). Adriana Tavares and Tashfeen Walton are funded by the British Heart Foundation (RG/16/10/32375 and FS/19/34/34354). Mark MacAskill is funded by the British Heart Foundation (PG/16/12/32022, PG/17/83/33370, and RG/16/10/32375), and Agne Stadulyte is funded by the British Heart foundation (RE/13/3/30183). David Newby is funded by the British Heart Foundation (CH/09/002, RG/16/10/32375, and RE/18/5/34216) and is the recipient of a Wellcome Trust Senior Investigator Award (WT103782AIA). Carlos Alcaide-Corral and Christophe Lucatelli are supported by the Edinburgh Preclinical Imaging and Edinburgh Imaging core facilities, respectively. The research team acknowledges the financial support of NHS Research Scotland (NRS), through Edinburgh Clinical Research Facility. No other potential conflict of interest relevant to this article was reported.

**KEY POINTS**

**QUESTION:** Is our newly developed TSPO radiotracer, ^18^F-LW223, able to detect macrophage-driven inflammation after MI while being suitable for clinical translation and not affected by the rs6971 human genetic polymorphism?
**PERTINENT FINDINGS:** Binding of ^18^F-LW223 to TSPO in human brain and heart in vitro assays demonstrated that it was not susceptible to the rs6971 genetic polymorphism, and the preclinical in vivo characterization indicated that ^18^F-LW223 is specific, has favorable metabolism, and has a dosimetry profile suitable for translation. In a preclinical rat MI model, ^18^F-LW223 BP_TC_ was significantly increased within the infarct relative to naïve myocardium, mirroring the pattern of macrophage expression in these animals detected by ex vivo immunofluorescent staining.
**IMPLICATIONS FOR PATIENT CARE:** As ^18^F-LW223 is not affected by the rs6971 human genetic polymorphism and has excellent in vivo characteristics, it is a prime candidate for further clinical translation that could ultimately lead to the development of a prognostic tool for use in patients after MI.
