# Supplementary material for: Quantification of Macrophage-Driven Inflammation During Myocardial Infarction with 18F-LW223, a Novel TSPO Radiotracer with Binding Independent of the rs6971 Human Polymorphism
Source: J Nucl Med. 2021 Apr;62(4):536–44. doi: 10.2967/jnumed.120.243600 (PMC8049364; doi:10.2967/jnumed.120.243600)
Supplement: Supplementary file 1 [file jnm243600SupplementaryData.pdf]

SUPPLEMENTAL METHODS

Organic Chemistry

All reagents and starting materials were obtained from commercial sources and used as received. All dry solvents were purified using a PureSolv 500 MD solvent purification system. All reactions were performed under argon unless otherwise stated. Brine is defined as a saturated solution of aqueous sodium chloride. Flash column chromatography was carried out using Fisher Matrix silica 60. Macherey–Nagel aluminium–backed plates pre–coated with silica gel 60 (UV254) were used for thin layer chromatography and were visualized using UV light. ^{1}H NMR and ^{13}C NMR spectra were recorded on a Bruker DPX 400 spectrometer or Bruker 500 spectrometer with chemical shift values in ppm relative to tetramethylsilane (δ_H 0.00 and δ_C 0.0) or residual chloroform (δ_H 7.26 and δ_C 77.2) as the standard. ^{1}H and ^{13}C assignments are based on two-dimensional COSY and DEPT experiments, respectively. Infrared spectra were recorded on a JASCO FTIR 410 spectrometer. Mass spectra were recorded using electron impact, chemical ionization or fast atom bombardment techniques. HRMS spectra were recorded using a dual-focusing magnetic analyzer mass spectrometer. Melting points were determined on a Gallenkamp melting point apparatus. Chiral High Performance Liquid Chromatography (HPLC) methods were calibrated with the corresponding racemic mixtures. 3-Methyl-4-phenylquinoline-2-carboxylic acid was prepared as previously reported *(14)*.

(*R*)-(*N*-*sec*-Butyl)-3-methyl-4-phenylquinoline-2-carboxamide

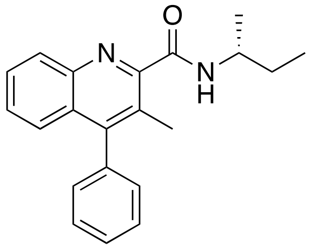

To a solution of 3-methyl-4-phenylquinoline-2-carboxylic acid (2.54 g, 9.65 mmol) in anhydrous *N*,*N*-dimethylformamide (250 mL) was added *O*-(benzotriazol-1-yl)-*N*,*N*,*N′*,*N′*-tetramethyluronium hexafluorophosphate (5.49 g, 14.5 mmol) and *N*,*N*-diisopropylethylamine (3.40 mL, 19.3 mmol). The reaction mixture was stirred at room temperature for 0.5 h before the addition of (*R*)-(−)-*sec*-butylamine (1.10 mL, 10.6 mmol) and then heated to 40 °C for 4 h. The reaction mixture was cooled to room temperature, diluted with ethyl acetate (300 mL) and washed with water (3 × 200 mL) and brine (200 mL). The organic layer was dried ($MgSO_4$), filtered and concentrated *in vacuo* to give a brown oil. Purification by flash column chromatography (petroleum ether/ethyl acetate, 4:1) gave (*R*)-(*N*-*sec*-butyl)-3-methyl-4-phenylquinoline-2-carboxamide as a white solid (2.81 g, 91%). Mp 152–154 °C (lit.*(15)* mp 157–158 °C); IR (KBr) 3287 (NH), 2968 (CH), 1641 (CO), 1539, 1448, 1157, 761 cm^{-1}; $[\alpha]_D^{25}$ –26.7 (*c* 1.0, $CHCl_3$); ^{1}H NMR (400 MHz, $CDCl_3$) δ 1.03 (3H, t, *J* = 7.4 Hz, $CHCH_2CH_3$), 1.33 (3H, d, *J* = 6.6 Hz, $CHCH_3$), 1.61–1.75 (2H, m, CH_2CH_3), 2.56 (3H, s, 3-CH_3), 4.08–4.20 (1H, m, $CHCH_3$), 7.21–7.25 (2H, m, ArH), 7.35 (1H, d, *J* = 8.3 Hz, ArH), 7.40–7.55 (4H, m, ArH), 7.65 (1H, ddd, *J* = 8.3, 6.8, 1.4 Hz, ArH), 7.90 (1H, d, *J* = 8.3 Hz, NH), 8.09 (1H, d, *J* = 8.3 Hz, ArH); ^{13}C NMR (101 MHz, $CDCl_3$) δ 10.6 (CH_3), 17.6 (CH_3), 20.5 (CH_3), 29.9 (CH_2), 46.8 (CH), 126.1 (CH), 127.5 (CH), 127.9 (CH), 128.6 (C), 128.6 (2 × CH), 128.7 (CH and C), 129.3 (2 × CH), 129.5 (CH), 137.3 (C), 144.7 (C), 149.5 (C), 150.1 (C), 166.2 (C); MS (CI) *m/z* 319

(M+H$^+$, 100%), 220 (19), 202 (5), 148 (6), 113 (16), 85 (77); HRMS (CI) calcd for $C_{21}H_{23}N_2O$ (M+H$^+$), 319.1810, found 319.1809.

(*R*)-(*N*-*sec*-Butyl)-*N*-methyl-3-methyl-4-phenylquinoline-2-carboxamide

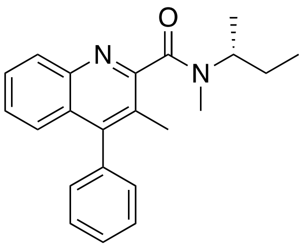

To a solution of (*R*)-(*N*-*sec*-butyl)-3-methyl-4-phenylquinoline-2-carboxamide (2.81 g, 8.82 mmol) in tetrahydrofuran (176 mL) was added sodium hydride (60% dispersion in mineral oil, 0.710 g, 17.6 mmol). The mixture was stirred at room temperature for 0.5 h, before the addition of iodomethane (2.75 mL, 44.1 mmol). The resultant solution was stirred at room temperature for 3 h and then quenched by addition of water. The aqueous phase was extracted with diethyl ether (3 × 10 mL). The combined organic phases were washed with a 10% aqueous solution of sodium thiosulfate (10 mL), brine (10 mL), dried (Na_2SO_4), filtered and concentrated *in vacuo*. Purification by flash column chromatography (petroleum ether/ethyl acetate, 3:1) gave (*R*)-(*N*-*sec*-butyl)-*N*-methyl-3-methyl-4-phenylquinoline-2-carboxamide as a white solid (2.75 g, 94%). NMR spectra showed a 1:1 mixture of rotamers. Signals for both rotamers are recorded. Mp 114–117 °C (lit.*(15)* mp 117–118 °C); IR (KBr) 2969 (CH), 1637 (CO), 1466, 1072, 731 cm^{-1}; $[\alpha]_D^{23}$ –6.3 (*c* 1.0, $CHCl_3$); ^{1}H NMR (400 MHz, $CDCl_3$) δ 0.86 (3H, t, *J* = 7.3 Hz, CH_2CH_3), 1.03 (3H, t, *J* = 7.3 Hz, CH_2CH_3), 1.24 (3H, d, *J* = 6.6 Hz, $CHCH_3$), 1.28 (3H, d, *J* = 6.6 Hz, $CHCH_3$), 1.36–1.71 (4H, m, 2 × CH_2CH_3), 2.21 (3H, s, 3-CH_3), 2.23 (3H, s, 3-CH_3), 2.73 (3H, s, NCH_3), 3.04 (3H, s, NCH_3), 3.42–3.53 (1H, m, $CHCH_3$), 4.84–4.94 (1H, m,

$CHCH_3$), 7.25–7.31 (4H, m, ArH), 7.38–7.44 (4H, m, ArH), 7.45–7.57 (6H, m, ArH), 7.60–7.67 (2H, m, ArH), 8.09 (1H, d, *J* = 8.3 Hz, ArH), 8.11 (1H, d, *J* = 8.3 Hz, ArH); ^{13}C NMR (101 MHz, $CDCl_3$) δ 11.2 (CH_3), 11.3 (CH_3), 16.0 (CH_3), 16.4 (CH_3), 17.3 (CH_3), 18.6 (CH_3), 25.5 (CH_3), 26.5 (CH_3), 27.2 (CH_2), 29.3 (CH_2), 49.6 (CH), 55.8 (CH), 124.6 (C), 125.3 (C), 125.9 (CH), 126.0 (CH), 126.7 (2 × CH), 126.8 (2 × CH), 127.4 (2 × C), 128.0 (2 × CH), 128.6 (2 × CH), 128.7 (2 × CH), 129.2 (4 × CH), 129.4 (2 × CH), 136.7 (C), 136.8 (C), 145.8 (C), 146.1 (C), 148.0 (C), 148.1 (C), 156.1 (C), 156.6 (C), 169.4 (C), 169.7 (C); MS (CI) *m/z* 333 ($M+H^+$, 100%), 291 (48), 250 (41), 220 (14), 86 (23); HRMS (CI) calcd for $C_{22}H_{25}N_2O$ ($M+H^+$), 333.1967, found 333.1972.

(*R*)-3-Bromomethyl-(*N*-*sec*-butyl)-*N*-methyl-4-phenylquinoline-2-carboxamide

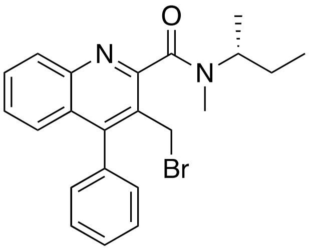

To a stirred, degassed solution of (*R*)-(*N*-*sec*-butyl)-*N*-methyl-3-methyl-4-phenylquinoline-2-carboxamide (2.70 g, 8.12 mmol) in chloroform (300 mL) was added *N*-bromosuccinimide (2.17 g, 12.2 mmol) and dibenzoyl peroxide (0.20 g, 0.812 mmol) and the solution heated under reflux for 6 h. A further portion of *N*-bromosuccinimide (1.00 g, 5.61 mmol) was then added and the solution heated under reflux for a further 16 h. The reaction mixture was cooled to room temperature, filtered and the solvent removed *in vacuo*. The crude residue was then diluted with ethyl acetate (100 mL) and washed with water (3 × 100 mL). The organic layer was dried ($MgSO_4$), filtered and concentrated *in vacuo*. Purification by flash column chromatography using a graduated eluent of

dichloromethane > dichloromethane/ethyl acetate (95:5) afforded (*R*)-3-bromomethyl-(*N*-*sec*-butyl)-*N*-methyl-4-phenylquinoline-2-carboxamide as an orange solid (2.8 g, 85%). NMR spectra showed a 2:1 mixture of rotamers. Only signals for the major rotamer are recorded. Mp 160–164 °C; IR (KBr) 2970 (CH), 1631 (CO), 1484, 1397, 1046, 766 cm^{-1}; $[\alpha]_D^{28}$ –9.0 (*c* 1.0, $CHCl_3$); ^1H NMR (400 MHz, $CDCl_3$) δ 1.09 (3H, t, *J* = 7.4 Hz, CH_2CH_3), 1.32 (3H, d, *J* = 6.8 Hz, $CHCH_3$), 1.51–1.80 (2H, m, CH_2CH_3), 2.86 (3H, s, NCH_3), 4.60 (1H, d, *J* = 10.2 Hz, 3-C*H*H), 4.67 (1H, d, *J* = 10.2 Hz, 3-CH*H*), 4.87 (1H, sextet, *J* = 6.8 Hz, C*H*CH_3), 7.37–7.48 (4H, m, ArH), 7.51–7.59 (3H, m, ArH), 7.70 (1H, ddd, *J* = 8.3, 6.7, 1.5 Hz, ArH), 8.10 (1H, dd, *J* 8.8, 8.3 Hz, ArH); ^{13}C NMR (101 MHz, $CDCl_3$) δ 11.1 (CH_3), 17.1 (CH_3), 26.6 (CH_2), 27.7 (CH_2), 30.5 (CH_3), 50.1 (CH), 126.3 (C), 126.7 (2 × CH), 127.4 (CH), 128.6 (2 × CH), 128.7 (CH), 129.0 (CH), 129.1 (CH), 129.5 (CH), 130.1 (C), 134.9 (C), 146.4 (C), 149.3 (C), 156.0 (C), 168.4 (C); MS (EI) *m/z* 410 (M^+, 5%), 298 (15), 296 (14), 217 (57), 189 (28), 151 (10), 86 (100); HRMS (EI) calcd for $C_{22}H_{23}{}^{79}BrN_2O$ (M^+), 410.0994, found 410.0992.

(*R*)-(*N*-*sec*-Butyl)-3-chloromethyl-*N*-methyl-4-phenylquinoline-2-carboxamide

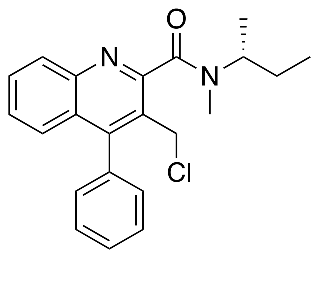

To a solution of (*R*)-3-bromomethyl-(*N*-*sec*-butyl)-*N*-methyl-4-phenylquinoline-2-carboxamide (0.500 g, 1.22 mmol) in dry tetrahydrofuran (10 mL) was added lithium chloride (0.160 g, 3.66 mmol) and the reaction mixture stirred at room temperature for 16 h. The reaction was quenched with water (30 mL) and extracted into ethyl acetate (3 ×

30 mL). The organic layers were combined and washed with brine (90 mL), dried ($MgSO_4$), filtered and concentrated *in vacuo*. The product was purified by flash column chromatography (dichloromethane/ethyl acetate, 95:5) to afford (*R*)-3-chloromethyl-(*N*-*sec*-butyl)-*N*-methyl-4-phenylquinoline-2-carboxamide as a white solid (0.331 g, 74%). NMR spectra showed a 1.5:1 mixture of rotamers. Only signals for the major rotamer are recorded. Mp 140–142 °C; IR (neat) 2970 (CH), 1620 (CO), 1481, 1404, 1219, 748 cm^{-1}; $[\alpha]_D^{24}$ –11.6 (*c* 1.0, $CHCl_3$); ^1H NMR (400 MHz, $CDCl_3$) δ 1.08 (3H, t, *J* = 7.4 Hz, CH_2CH_3), 1.30 (3H, d, *J* = 6.8 Hz, $CHCH_3$), 1.49–1.79 (2H, m, CH_2CH_3), 2.84 (3H, s, NCH_3), 4.67 (1H, d, *J* = 10.6 Hz, 3-C*H*H), 4.72 (1H, d, *J* = 10.6 Hz, 3-CH*H*), 4.82–4.92 (1H, m, C*H*CH_3), 7.36–7.61 (7H, m, ArH), 7.69–7.75 (1H, m, ArH), 8.11 (1H, dd, *J* 9.0, 8.4 Hz, ArH); ^{13}C NMR (101 MHz, $CDCl_3$) δ 11.1 (CH_3), 17.1 (CH_3), 26.6 (CH_2), 30.4 (CH_3), 40.4 (CH_2), 50.1 (CH), 125.8 (C), 126.8 (CH), 127.2 (C), 127.4 (CH), 128.5 (2 × CH), 128.7 (CH), 129.4 (CH), 129.6 (2 × CH), 130.1 (CH), 134.9 (C), 146.6 (C), 149.5 (C), 156.1 (C), 168.5 (C); MS (ESI) *m/z* 389 ($M+Na^+$, 100%); HRMS (ESI) calcd for $C_{22}H_{23}{}^{35}ClN_2NaO$ ($M+Na^+$), 389.1391, found 389.1381.

(*R*)-(*N*-*sec*-Butyl)-3-fluoromethyl-*N*-methyl-4-phenylquinoline-2-carboxamide

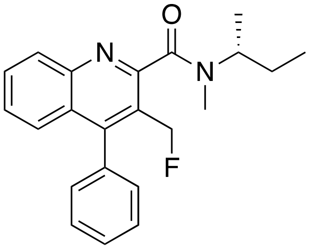

To a solution of 18-crown-6 (0.032 g, 0.12 mmol) in acetonitrile (2.5 mL) was added potassium fluoride (0.036 g, 0.61 mmol) and the resulting suspension stirred at room temperature for 0.5 h. A solution of (*R*)-3-bromomethyl-(*N*-*sec*-butyl)-*N*-methyl-4-

phenylquinoline-2-carboxamide (0.050 g, 0.12 mmol) in acetonitrile:dichloromethane (2:1, 9.0 mL) was then added dropwise and the reaction mixture heated under reflux for 72 h. Upon completion, the reaction mixture was cooled to ambient temperature and water (20 mL) was added. The solution was extracted with dichloromethane (3 × 20 mL), dried ($MgSO_4$), filtered and concentrated *in vacuo*. Purification by flash column chromatography (petroleum ether/ethyl acetate 7:3) gave (*R*)-*N*-(*sec*-butyl)-3-(fluoromethyl)-*N*-methyl-4-phenylquinoline-2-carboxamide as a white solid (0.029 g, 67%). NMR spectra showed a 3:1 mixture of rotamers. Only signals for the major rotamer are recorded. Mp 146–148 °C; IR (neat) 2972 (CH), 1628 (CO), 1559, 1485, 1398, 1049, 970 cm^{-1}; $[\alpha]_D^{30}$ –12.6 (*c* 0.5, $CHCl_3$); ^1H NMR (400 MHz, $CDCl_3$) δ 1.05 (3H, t, J = 7.4 Hz, CH_2CH_3), 1.29 (3H, d, J = 6.8 Hz, $CHCH_3$), 1.41–1.78 (2H, m, CH_2CH_3), 2.77 (3H, s, NCH_3), 4.84–4.95 (1H, m, NC*H*), 5.31 (1H, dd, J 20.8, 10.8 Hz, 3-C*H*H), 5.44 (1H, dd, J 20.8, 10.8 Hz, 3-CH*H*), 7.31–7.40 (2H, m, ArH), 7.43–7.58 (5H, m, ArH), 7.74 (1H, t, J 7.6 Hz, ArH), 8.17 (1H, d, J 8.4 Hz, ArH); ^{13}C NMR (101 MHz, $CDCl_3$) δ 10.9 (CH_3), 17.4 (CH_3), 26.5 (CH_3), 29.9 (CH_2), 50.0 (CH), 79.2 (CH_2, $^1J_{C\text{–}F}$ = 162.8 Hz), 123.0 (C, $^2J_{C\text{–}F}$ = 15.1 Hz), 127.0 (CH), 127.1 (C, $^4J_{C\text{–}F}$ = 2.3 Hz), 127.4 (CH, $^5J_{C\text{–}F}$ = 1.2 Hz), 128.5 (2 × CH), 128.7 (CH), 129.6 (2 × CH), 129.7 (CH), 130.4 (CH), 134.8 (C, $^4J_{C\text{–}F}$ = 1.5 Hz), 147.4 (C, $^3J_{C\text{–}F}$ = 2.5 Hz), 150.8 (C, $^3J_{C\text{–}F}$ = 4.7 Hz), 156.6 (C, $^5J_{C\text{–}F}$ = 2.1 Hz), 168.8 (C); MS (ESI) *m/z* 373 ($M+Na^+$, 100%); HRMS (ESI) calcd for $C_{22}H_{23}FN_2NaO$ ($M+Na^+$), 373.1687, found 373.1670. Enantiomeric excess was determined by HPLC analysis with a chiralcel AD-H column (hexane:^{i}PrOH 97.5:2.5, flow rate 1.0 mL/min), t_{major} = 30.68 and 32.22 min, t_{minor} = 27.15 and 38.38 min; er = 99.5:0.5.

In Vitro Human Competition Binding Assays

Briefly, 250 µg protein/mL in 200 µl of buffer was added to 100 µl of 1 nM ^{3}H-PK11195 (PerkinElmer, USA), together with 100 µl of test ligands PK11195 (Sigma-Aldrich, USA), PBR28 (ABX, Germany), AB5186 or LW223 at 14 different concentrations (0.001-3000 nM) for 90 min at 4 ºC. Binding was terminated with the addition of ice cold buffer before being immediately filtered over a Whatman GF/B filters (Whatman, UK) pre-treated with 0.3% polyethylenimine (Sigma-Aldrich, USA) using a Brandel harvester (Brandel, USA). Filter paper was then removed, placed into 2.5 mL of Optiphase HiSafe 3 (Perkin Elmer, USA) and counted 48 hours later on a Hidex 300 SL (Hidex, Finland). Saturation assays were performed to determine the K_d of PK11195 using a similar protocol, except that 6 concentrations of ^{3}H-PK11195 (1.6-200 nM) were used alongside 10 µM of PK11195 to determine non-specific binding. All binding assays were performed in triplicate.

GraphPad Prism version 6 (GraphPad Software, USA) was used to fit all binding affinity curves. A comparison of a one-site and two-site models was made using the least squares algorithm and model selection was compared using an F test. The null hypothesis (one-site fitting as more suitable) was rejected if $p<0.05$. Normalized mean %SB of each group (HAB, MAB or LAB) and a K_d value of 14.0nM (determined based on saturation binding results, Sup.Fig.2) was used to determine the best fitting model and to calculate LAB:HAB ratios. Affinity values (K_i) were calculated by fitting individual tissue samples again with a K_d 14.0nM.

Invasive Input Function Measurement

Polyethylene catheters (PE50) filled with heparinized saline (20 IU/mL) were inserted into the left femoral artery and vein with the help of a stereomicroscope and securely fastened with ligatures (6-0 silk thread). Catheters were held in place with surgical glue.

Rat Myocardial Infarction Procedure

Briefly, the skin was incised at the level of the left third and fourth ribs where the pectoral muscles were divided and retracted. Left lateral thoracotomy was then performed. With minimal handling, the pericardium was ruptured and the heart gently exteriorized from the thorax, and a nonabsorbable 5-0 ligature was placed around the left anterior descending coronary artery just above the bifurcation of the first diagonal and maneuvered back into position. Before wound closure, a drain was inserted to assist with removal of air and fluid from the thorax. Once removed, the wound was then closed in three layers. Animals were recovered and extubated once spontaneous ventilation was established, housed at 30 °C for 24 hours and given sterile sodium chloride 0.9% 0.01 mL/g fluid therapy subcutaneously in addition to another dose of Buprenorphine. After 24 hours, normal housing conditions were resumed.

Radiometabolite Blood and Tissue Processing and Analysis

All blood samples were 1 mL each and manually collected from different animals to generate a population curve, in order to respect total blood volume limits for terminal arterial blood collections in rats. Following blood and tissue collection, all samples were

kept on ice until analyzed. Radioactivity in whole blood and plasma, as well as homogenized tissue samples, was assessed using a well-type γ-counter using a 400–1400 keV window (Perkin Elmer Wizzard2, USA). Plasma samples (400 μl) and tissue samples (500 μl) were processed by acetonitrile denaturation (1:1.4 v/v) and analyzed by HPLC (Ultimate2000, ThermoFisher, UK) on a Luna C18(2) 10×250 mm, 10 μm column (Phenomenex, UK) with acetonitrile/water 70/30 and flow rate of 4 mL/min to estimate the parent fraction. The plasma f_p was determined using ultrafiltration units (Centrifree® 30K, Millipore, UK).

Simulation of Changes in ^{18}F-LW223 BP_{ND} for varying blood flow scenarios

Simulation work was carried out using a Matlab (MathWorks, USA) 2-tissue compartment code with Ordinary Differential Equation (ODE) solvers. Six different levels of K_1 values (0.9, 0.7, 0.5, 0.3, 0.1 and 0.01 mL/min/cc) were tested with fixed k_2 and vB=0.05. The following BP_{ND} values were simulated: 4, 10, 16 and 20; with k_3 modified and k_4 fixed. All micro parameters used in the simulations are shown in Supplemental Table 5. The input function used was an average of the blood curves taken from healthy animals using the SwissTrace blood sampler (Supplemental Data File 1). The plasma to whole blood ratio used was 1.84 and the AIF was metabolite corrected using a population based curve as in the experimental data. All modelled time-activity curves were noise free. Outcome measures were estimated using Turku code (fitk4, http://www.turkupetcentre.net/petanalysis/tpcclib/doc/fitk4.html) and operational equations for 2-tissue curve fitting. The minimisation code used Topographical Global Optimisation based on an algorithm by Aimo Torn and Sami Viitanen

(www.abo.fi/~atorn/ProbAlg/Page53.html). Calculated BP_{ND} for each simulated scenario was compared with truth BP_{ND}. All parameters estimated with the parameter standard deviation for K_1 and BP_{ND} are reported in Supplemental Table 6.

Ex Vivo **Immunofluorescence Imaging**

Briefly, antigen retrieval was carried out in a pressure cooker using tris-EDTA (pH 9) before an endogenous peroxidase block (3% H_2O_2, 15 min) and tissue permeabilization/ blocking in 10% donkey serum (PBS, 0.1% Triton X-100) for 1 hour room at temperature. The CD68 primary antibody (1/100 in 1% donkey serum, MCA341R, Bio-Rad, USA) was incubated with the slides overnight at 4 °C, followed by incubation with the first secondary antibody (1/750 in 1% donkey serum, donkey anti-mouse-HRP, 715-036-150, Jackson ImmunoResearch, USA) for 1 hour at room temperature. This first secondary antibody was visualized using the TSA system (Cyanine 3 System, 1/50 for 4 min, Perkin Elmer, UK). Microwave antigen retrieval (tris-EDTA pH 9) was then carried out to destroy the CD68-associated antibodies, leaving behind the visualization product only. An additional endogenous peroxidase and serum block was performed before incubation with the TSPO primary antibody (1/100 in 1% donkey serum, ab109497, Abcam, UK) for 1 hour at room temperature. A subsequent secondary antibody incubation was performed at 1 hour room temperature (1/750 in 1% donkey serum, donkey anti-rabbit-HRP, 711-036-152, Jackson ImmunoResearch, USA) before visualization with the TSA reagent (Cyanine 5 System, 1/50 for 4 min, Perkin Elmer, UK). The slides were then counterstained with DAPI and mounted.

Autoradiography

Rehydrated tissue on glass slides was incubated in assay buffer (50 mM Tris Buffer) for 30 min prior to the experiment. The slides were then incubated with ^{18}F-LW223 (50 nM in assay buffer) for 1 hour at room temperature. Slides were washed twice in assay buffer followed by a dip in $_{d}H_2O$. The slides were then dried and exposed to an autoradiography plate (BAS-IP-SR 2040 super resolution screen, Cytiva, USA) and imaged on an autoradiography imager (Amersham Typhoon IP Biomolecular Imager, Cytiva, USA). Images were analyzed using Image J, using the ratio of signal within a ROI to the background signal on each slide to calculate the tissue to background ratio (TBR).

SUPPLEMENTARY RESULTS

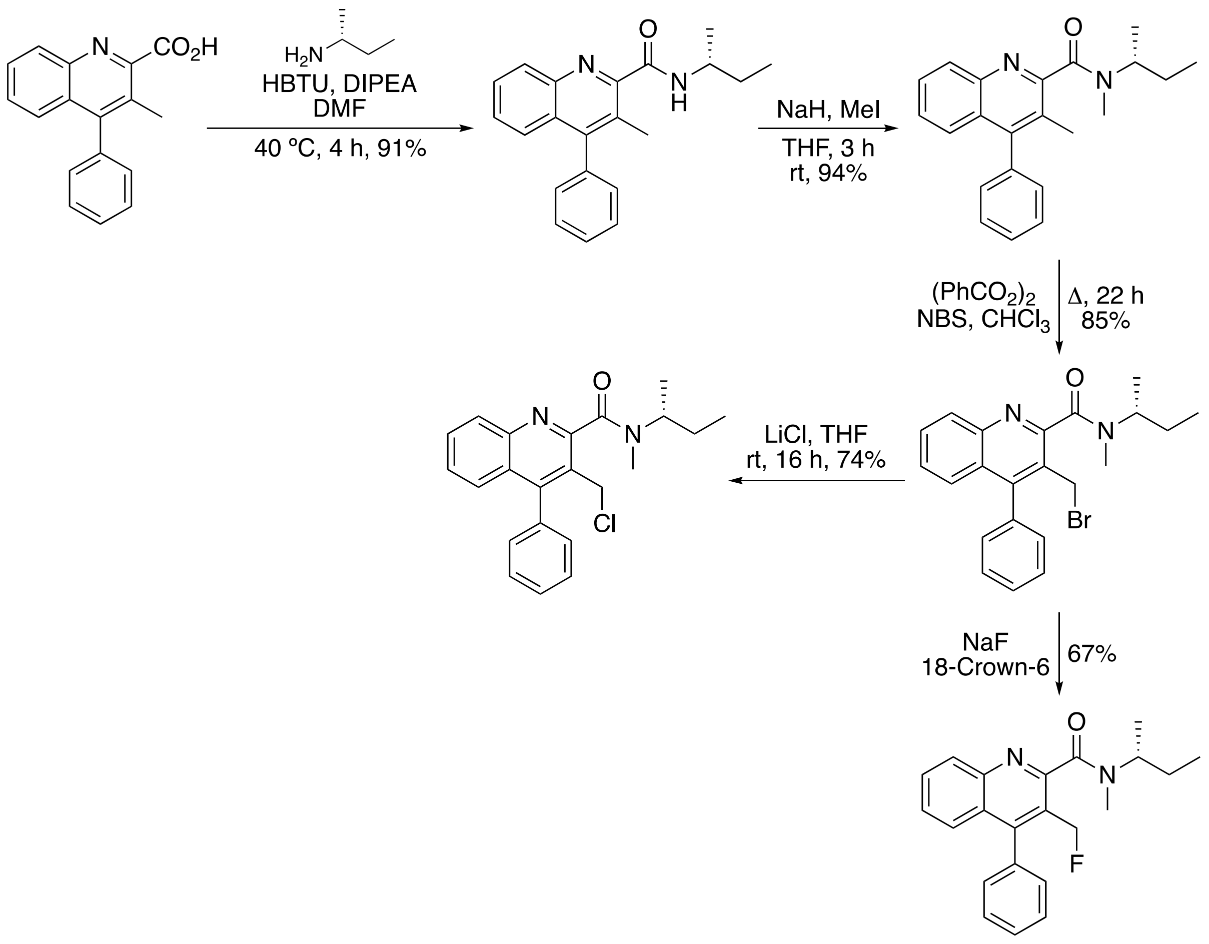

Supplemental Figure. 1. Four step synthesis of chloride precursor and LW223 from 3-methyl-4-phenylquinoline-2-carboxylic acid.

Shimadzu LCMS-2010 EV
Shimadzu LC2010A HT

Sample Name:	LW223
Method:	1.2 mL/min; 10–90% MeCN/0.1% Formic Acid(aq) – 20 min
Column:	Kinetex® 5μm XB-C18 100Å; LC Column 50 × 4.6 mm

LC Chromatogram:

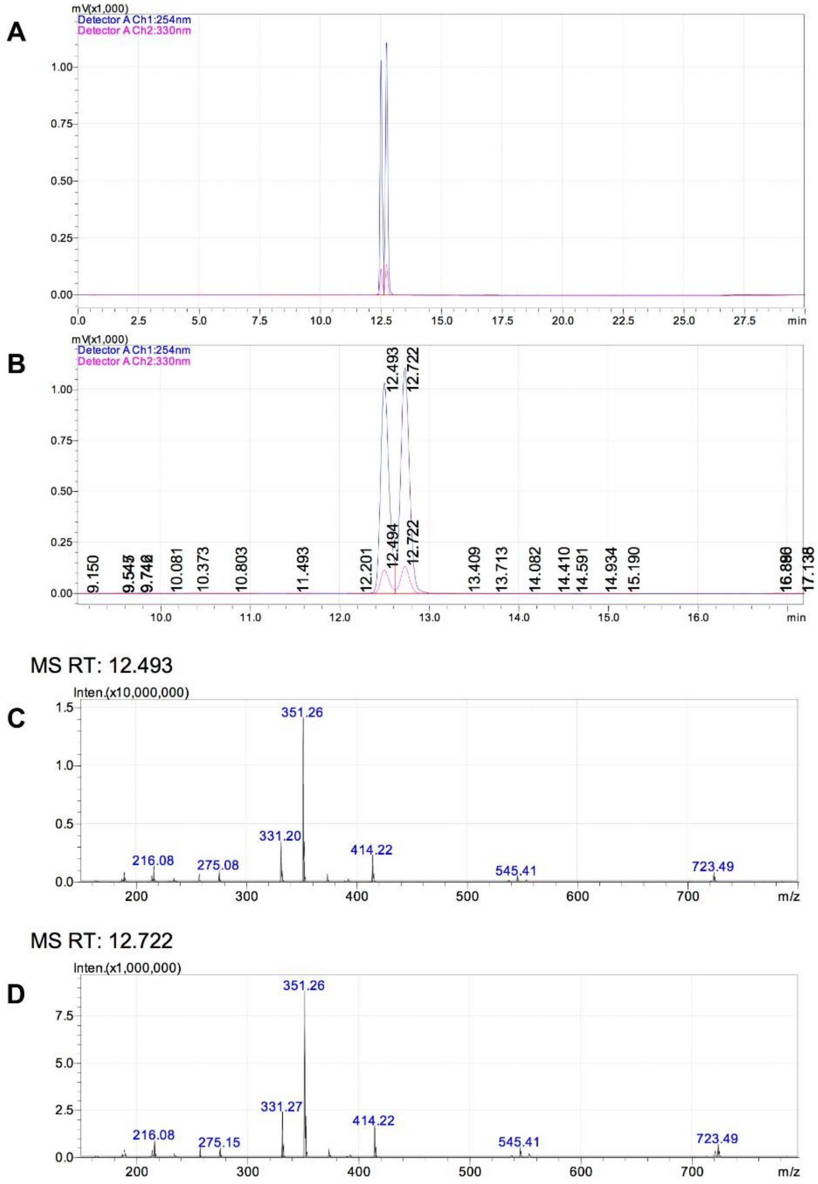

Supplemental Figure 2. HPLC and MS analysis of LW223 rotamers. A) LCMS trace of LW223 showing existence of two rotamers. **B)** Expansion of LC-MS trace of LW223. **C)** MS of first rotamer (12.493 minutes). **D)** MS of second rotamer (12.722 minutes).

Brain Tissue for Binding Assays	Heart Tissue for Binding Assays
25055	7626
24781	26124
24778	20592
22629	25055
21396	001.29466
18391	2580
9508	26309
4176	29084
13410	3784
2564	4176
3785	18393
3783	3786
2576	18392
2575	25751
2572	22611
2562	9508
2559	2473
2558	
2552	
2551	
2550	
2452	
24340	
22630	
22628	
22612	
20593	
20121	
18407	
15809	

Supplemental Table 1. Edinburgh Brain and Tissue Bank BNN reference number for all tissues utilized from the bank.

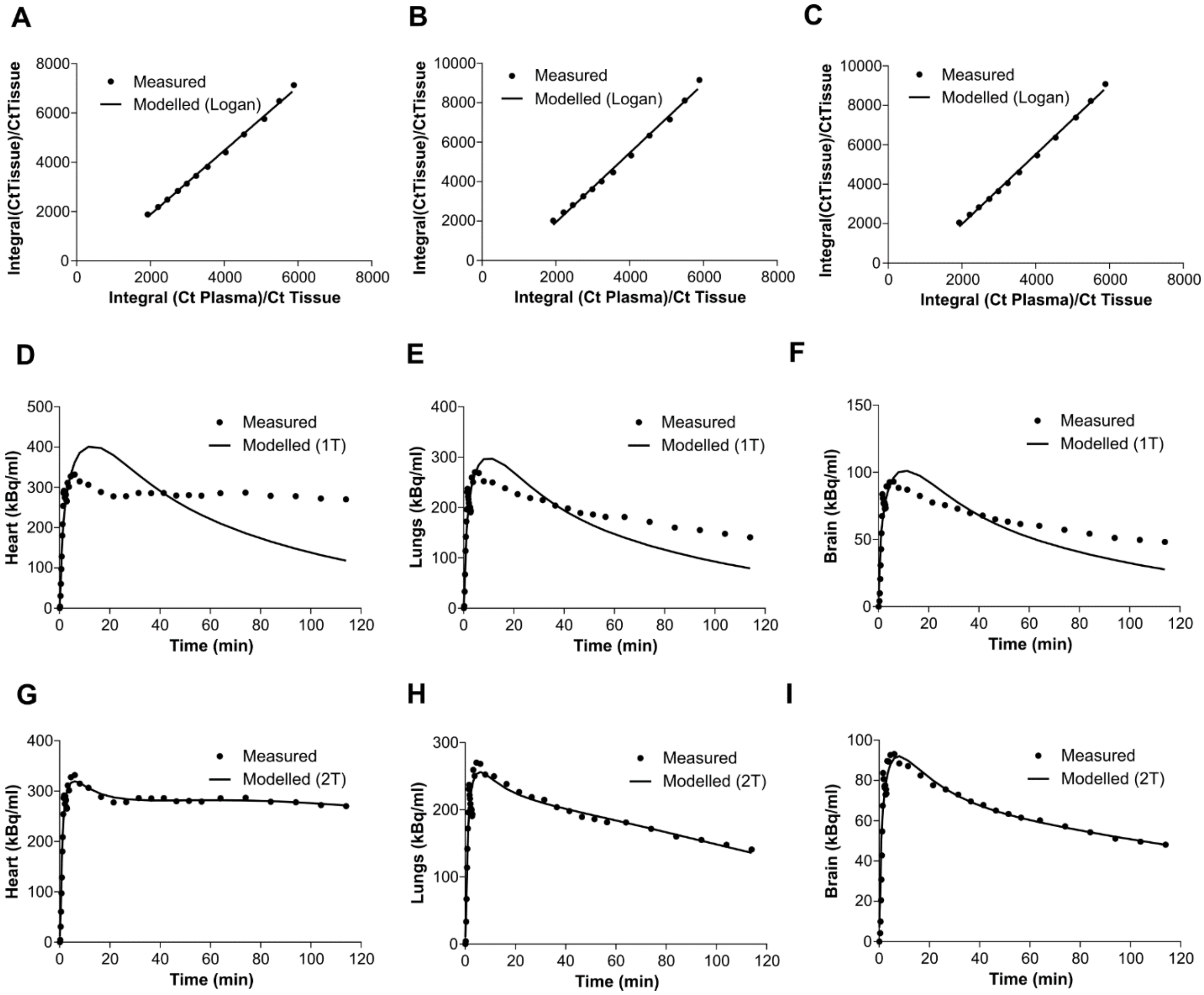

Supplemental Figure 3. Representative examples of different models versus measured data on naive rat organs. A) Logan modelling in the heart, **B)** lung and **C)** brain. Modelling constrained to a *t**=30min. **D)** 1T modelling in the heart, **E)** lung and **F)** brain. **G)** 2T modelling in the heart, **H)** lung and **I)** brain.

Region	Group	AIC (Mean± SEM)		
		2T	1T	Logan
Heart	Naive	47.8± 6.3	131.9± 2.7	125.8± 4.4
	MI	52.1± 4.2	135.5± 4.8	132.7± 1.6
	Blocked Naive	132± 27.3	110.2± 31.5	118.1± 6.3
LV Anterior Wall	Naive	127.9± 12.4	159.1± 6.2	144.5± 1.9
	MI	121.5± 8.9	143.7± 7.1	151.7± 2.1
	Blocked Naive	160.7± 15.5	136.6± 3.9	148.6± 5.1
Brain	Naive	42.8± 3.4	100.6± 4.8	117.1± 3.1
	MI	57.8± 12.9	118.8± 9.7	118.6± 3.5
	Blocked Naive	153.3± 18	128.9± 4.4	128.3± 5.3
Lungs	Naive	62.6± 5.7	105.4± 4.1	112.6± 2.8
	MI	60.7± 4	123.9± 7.8	120.7± 2.6
	Blocked Naive	156.5± 21.4	138.1± 1.8	119.6± 6.5
	Group Mean	98.0	127.8	128.2

Supplemental Table 2. The Akaike information criterion (AIC) for assessed models in all animals and regions used in this study, naive *n* = 6, MI *n* = 9, blocked naive *n* = 3.

Organ	Mouse<br>% Total body weight	Human<br>% Total body weight	Correction Factor
**Brain**	1.62	1.93	1.19
**Heart**	0.48	0.43	0.89
**Lung**	0.53	1.36	2.55
**Gall Bladder**	0.03	0.01	0.44
**Liver**	4.00	2.59	0.65
**Gut**	5.01	0.92	0.18
**Adrenals**	0.02	0.02	1.09
**Kidney**	1.28	0.41	0.32
**Bladder**	0.08	0.06	0.76

Supplemental Table 3. Scaling factors for different organs in mice and humans based on their percentage contribution to total body weight. Data presented in this table was calculated based on previously published data.

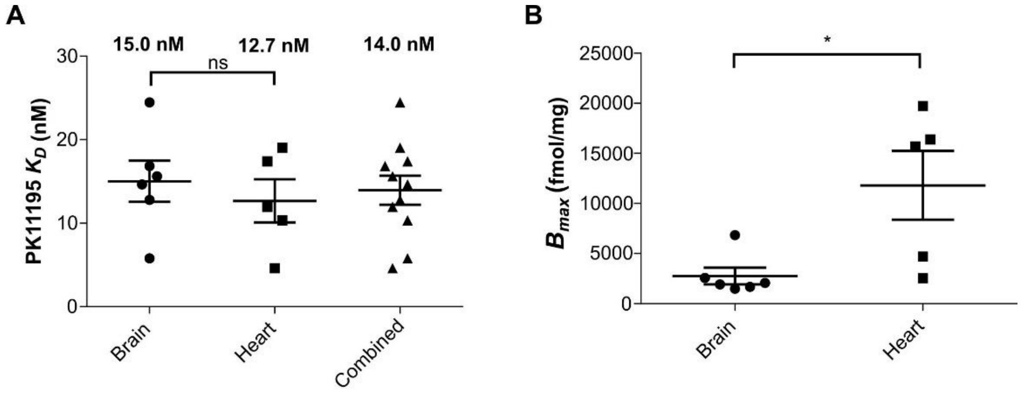

Supplemental Figure 4. Dissociation constant (*K_d*) values and maximal binding (*B_{max}*) of PK11195 in human brain and heart. A) K_d values of PK11195 and **B)** B_{max} calculated from saturation binding assays. Results represent the mean±S.E.M., brain n = 6, heart n = 5, ns=not significant, *=p<0.05 using an unpaired *t*-test for brain versus heart.

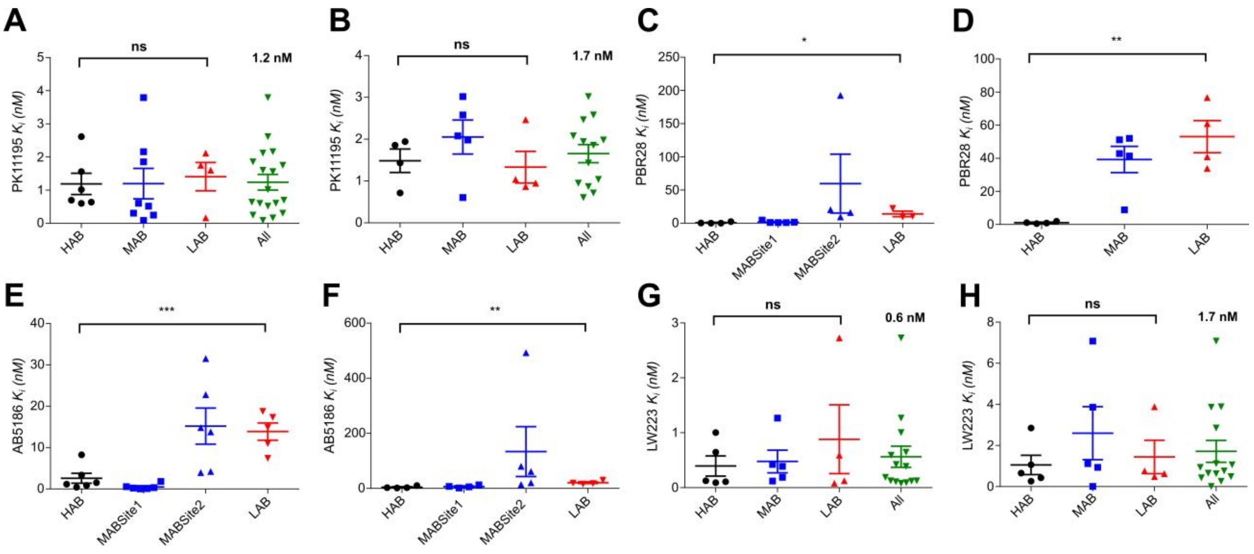

Supplemental Figure 5. Individually calculated K_i values for TSPO ligands using human brain and heart. A) PK11195 binding affinities in brain calculated using one-site fitting, HAB n = 6, MAB n = 8 and LAB n = 4 and **B)** heart calculated using a one-site fitting, HAB n = 4, MAB n = 5 and LAB n = 4. **C)** PBR28 binding affinities in brain calculated using a one-site fitting apart from MAB where a two-site fitting was used, HAB n = 4, MAB n = 5 and LAB n = 4 and **D)** heart calculated using a one-site fitting, HAB n = 4, MAB n = 5 and LAB n = 4. **E)** AB5186 binding affinities in brain calculated using a one-site fitting apart from MAB where a two-site fitting was used, HAB n = 6, MAB n = 6 and LAB n = 5 and **F)** heart using a one-site fitting apart from MAB where a two-site fitting was used, HAB n = 4, MAB n = 5 and LAB n = 4. **G)** LW223 binding affinities in brain calculated using a one-site fitting, HAB n = 5, MAB n = 5 and LAB n = 4 and **H)** heart calculated using a one-site fitting, HAB n = 5, MAB n = 5 and LAB n = 4. ns=not significant, *=$p<0.05$, **=$p\leq0.01$ and ***=$p\leq0.001$ using an unpaired t-test for HAB vs. LAB. All results represent the mean±SEM.

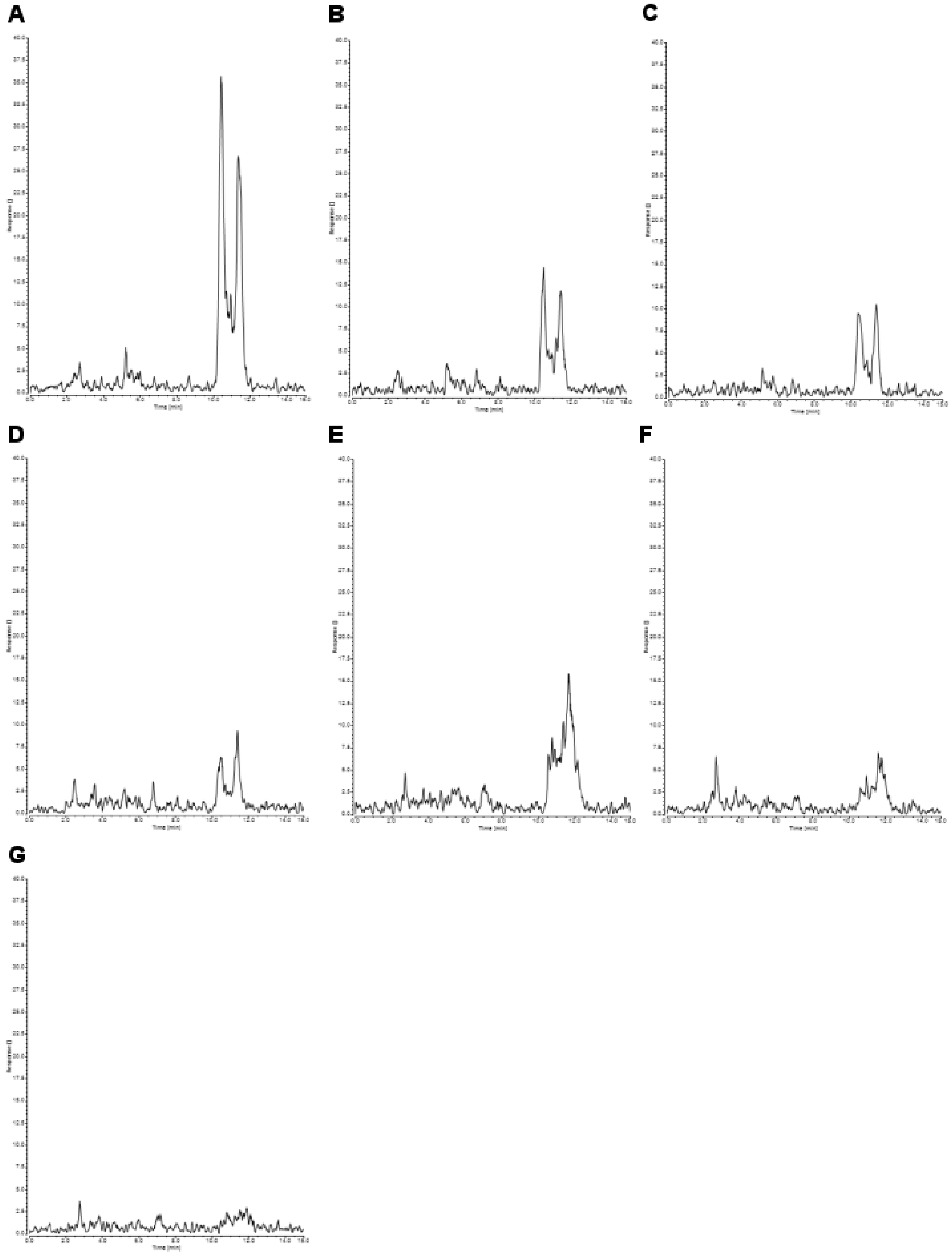

Supplemental Figure 6. Representative chromatograms of arterial blood sampled at 2-120 min post-injection of ^{18}F-LW223. A) Radiodetection of parent peak and radiometabolites in rat atrial plasma at 2 min, **B)** 5 min, **C)** 10 min, **D)** 20 min, **E)** 30 min, **F)** 60 min and **G)** 120 min post intravenous bolus administration of ^{18}F-LW223.

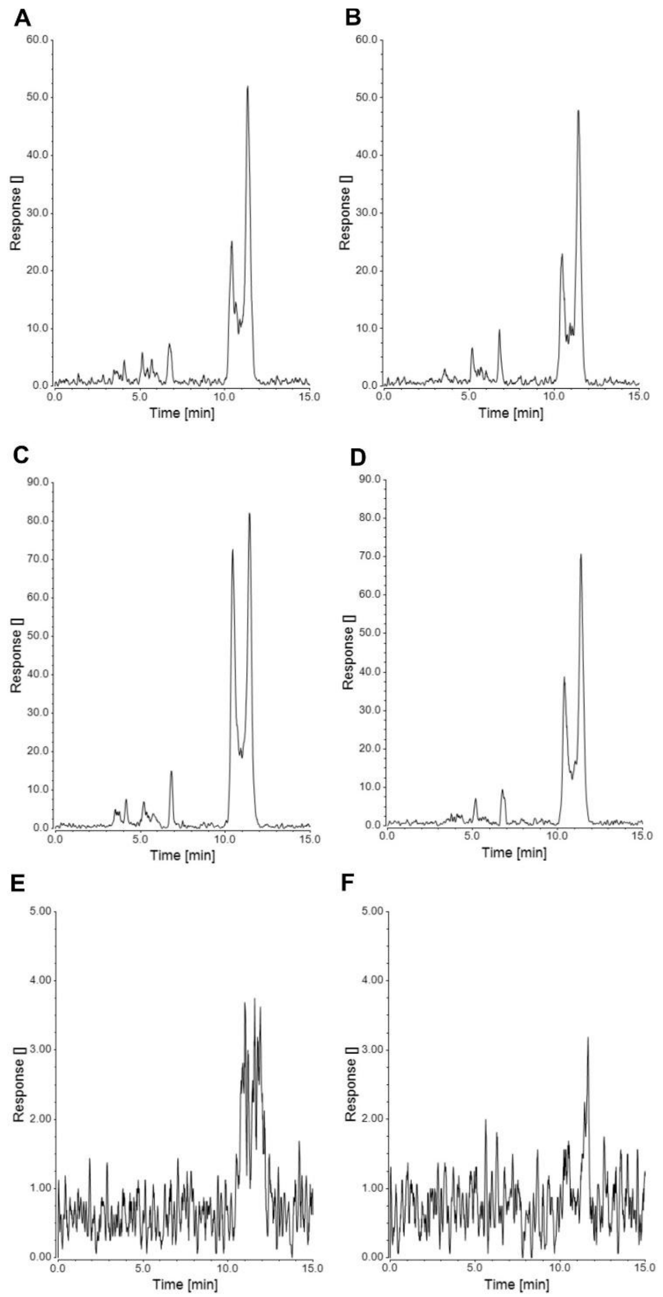

Supplemental Figure 7. Representative chromatograms of organs sampled at 60 and 120 min post-injection of ^{18}F-LW223. A) Radiodetection of parent peak and radiometabolites in rat heart at 60 min, **B)** heart at 120 min post injection, **C)** lung at 60 min, **D)** lung at 120 min, **E)** brain at 60 min and **F)** brain at 120 min post intravenous bolus administration of ^{18}F-LW223.

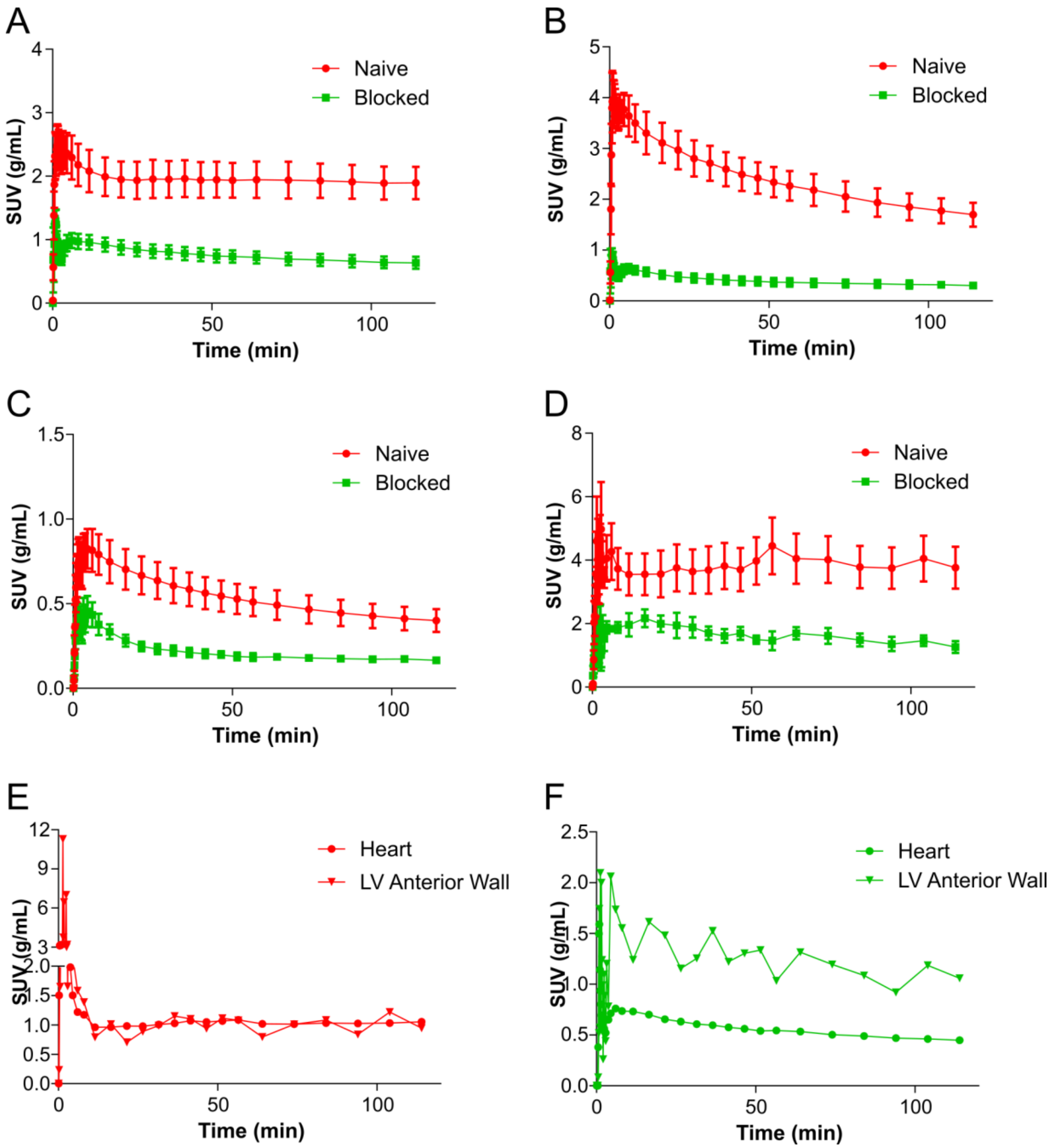

Supplemental Figure 8. SUV time activity curves in naive and naive blocking studies. A) The effect of PK11195 blockade (1 mg/kg) on ^{18}F-LW223 in the heart, **B)** lung, **C)** brain and **D)** sampled left ventricle (LV) anterior wall. Mean ±SEM, naive n = 6, blocked n = 3. **E)** Example time activity curves from a single naive and **F)** blocked naive animal demonstrating the noise which is present in the LV subregion sample.

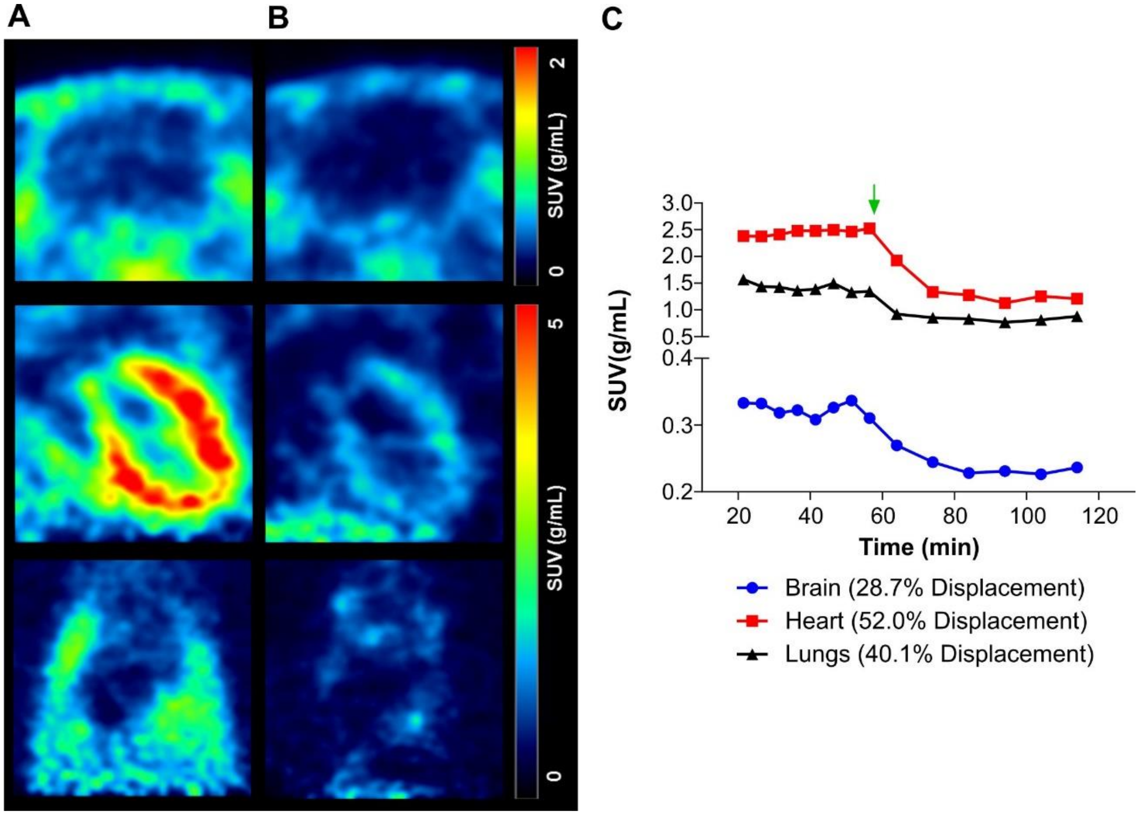

Supplemental Figure 9. *In vivo* displacement of ^{18}F-LW223 using PK11195 in naive rats. A) SUV sum image of ^{18}F-LW223 uptake before and after **B)** displacement with PK11195 (1mg/kg). Top row=brain, middle row=heart and bottom row=lung. Baseline images are averaged before (45-60 min) and displacement images are averaged after (95-120 min) PK11195 administration. **C)** Time activity curve of ^{18}F-LW223 before and after (green arrow) PK11195 challenge.

Region	Group	K1		k2		k3		k4		k3/k4		VT	
		Mean± S.E.M.	% SE± S.E.M	Mean± S.E.M.	% SE± S.E.M	Mean± S.E.M.	% SE± S.E.M	Mean± S.E.M.	% SE± S.E.M	Mean± S.E.M.	% SE± S.E.M	Mean± S.E.M.	% SE± S.E.M
Global Heart	Naive	0.58± 0.06	6.09± 0.99	0.33± 0.01	12.24± 1.51	0.031± 0.004	18.51± 2.04	0.01± 0.00	46.319± 9.764	4.77± 0.27	32.43± 7.72	9.93± 0.98	29.09± 6.83
	MI	0.58± 0.04	6.57± 0.71	0.36± 0.03	13.46± 1.07	0.04± 0.004	17.38± 1.46	0.01± 0.00	38.834± 5.141	6.62± 0.98	26.94± 4.09	12.57± 1.39	25.22± 4.06
	Blocked Naive	0.19± 0.01	68.38± 50.01	0.23± 0.02	464.35± 402.47	0.721± 0.638	66.85± 24.71	0.13± 0.11	475.458± 399.637	4.52± 0.66	472.01± 411.64	4.56± 0.22	23.00± 13.02
LV Anterior Wall	Naive	0.9± 0.11	15.67± 1.03	0.26± 0.03	36.50± 3.72	0.033± 0.005	59.73± 13.92	0.00± 0.00	224.581± 74.786	7.99± 1.21	176.71± 61.63	29.95± 3	164.47± 53.64
	MI	0.25± 0.03	13.95± 1.42	0.18± 0.02	36.24± 3.63	0.025± 0.003	71.52± 12.28	0.01± 0.00	267.21± 58.427	7.92± 1.31	216.05± 57.91	13.62± 3.21	201.04± 55.93
	Blocked Naive	0.28± 0.01	115.97± 106.93	0.08± 0.03	1463.29± 1425.50	0.855± 0.854	542.66± 354.24	0.23± 0.23	105717.505± 102167.414	12.64± 8.90	105197.46± 101812.77	75.25± 63.13	98934.28± 98923.61
Brain	Naive	0.14± 0.02	3.82± 0.21	0.18± 0.02	9.89± 0.58	0.016± 0.001	31.48± 2.63	0.01± 0.00	41.74± 3.31	1.22± 0.10	20.4± 2.51	1.6± 0.17	12.69± 2.08
	MI	0.17± 0.01	5.12± 0.69	0.19± 0.01	13.53± 1.84	0.023± 0.003	36.80± 6.29	0.02± 0.00	50.061± 13.8	1.42± 0.08	26.99± 7.8	2.21± 0.24	17.20± 5.84
	Blocked Naive	0.06± 0	471.22± 154.47	0.11± 0.02	18048.79± 12808.17	2.324± 1.818	15742.04± 3854.98	2.33± 1.03	20944.155± 15962.239	0.71± 0.32	36685.81± 19130.43	0.93± 0.02	21.85± 4.74
Lung	Naive	0.94± 0.06	6.51± 0.78	0.32± 0.02	13.99± 1.06	0.031± 0.007	32.87± 3.51	0.02± 0.00	38.458± 7.495	1.38± 0.13	20.15± 2.97	6.94± 0.51	10.89± 2.35
	MI	1.08± 0.17	7.24± 0.8	0.39± 0.07	15.09± 1.01	0.046± 0.011	24.35± 2.09	0.02± 0.00	26.524± 3	2.49± 0.34	15.17± 1.02	10.43± 1.60	9.36± 0.93
	Blocked Naive	0.16± 0.04	19± 5.43	0.35± 0.16	82.05± 23.02	0.181± 0.119	132.22± 30.37	0.05± 0.03	102.311± 49.984	3.19± 0.41	98.05± 26.56	2.2± 0.19	37.68± 22.85

Supplemental Table 4. Kinetic modelling data for 2T analysis of ^{18}F-LW223 uptake in naive, myocardial infarction (MI) and blocked rats (post injection of PK11195, 1mg/kg). Naive *n* = 6, MI *n* = 9, Blocked *n* = 3.

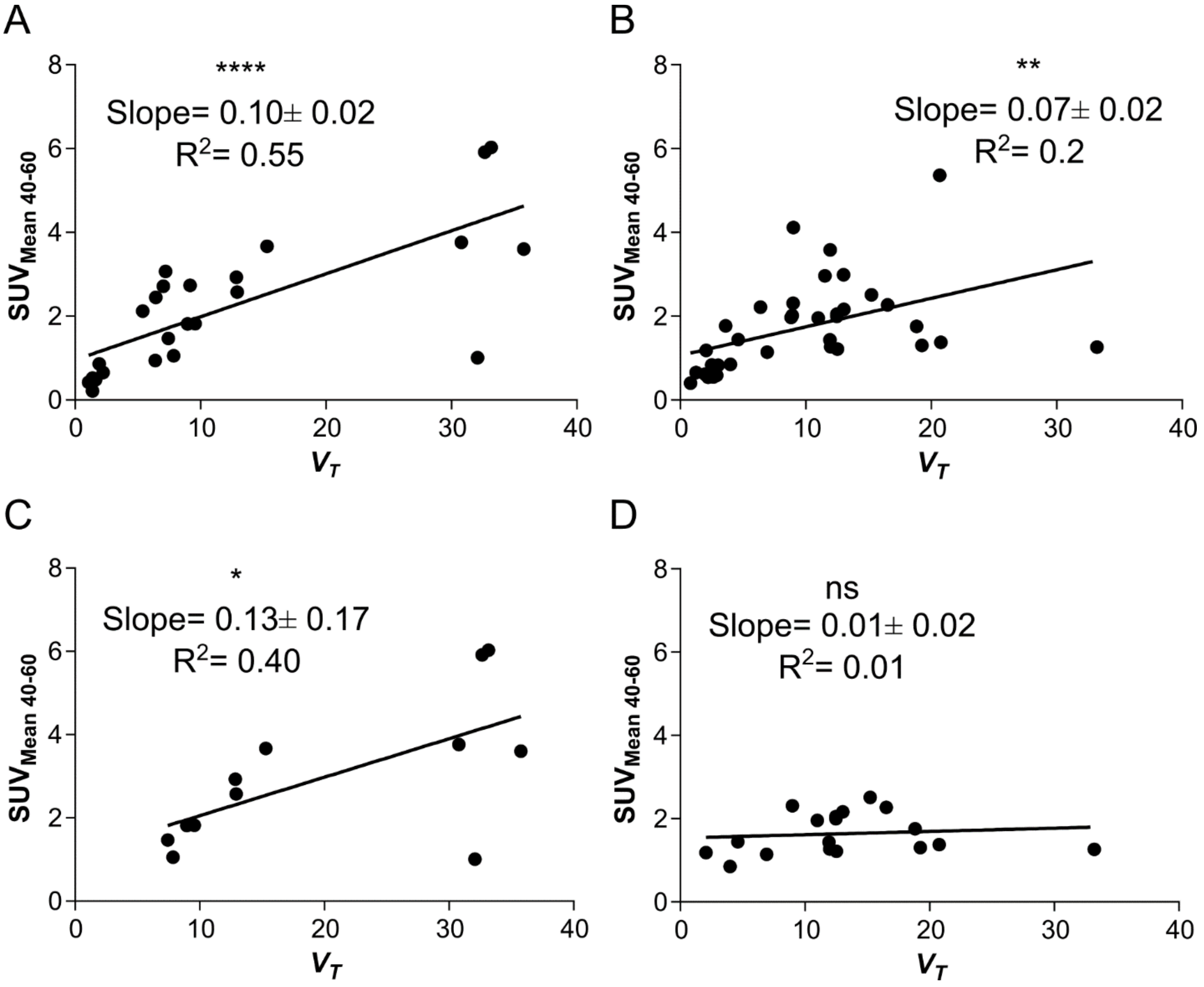

Supplemental Figure 10. Correlation of V_T and SUV in naive and MI animals. A) Global heart, left ventricle sample, brain and lung correlation in naive rats and **B)** MI rats. **C)** Global heart and left ventricle sample correlation in naive rats and **D)** MI rats. Naive *n* = 6, MI *n* = 9, *=p<0.05, **=p<0.01 and ****=p<0.0001 using Pearson correlation.

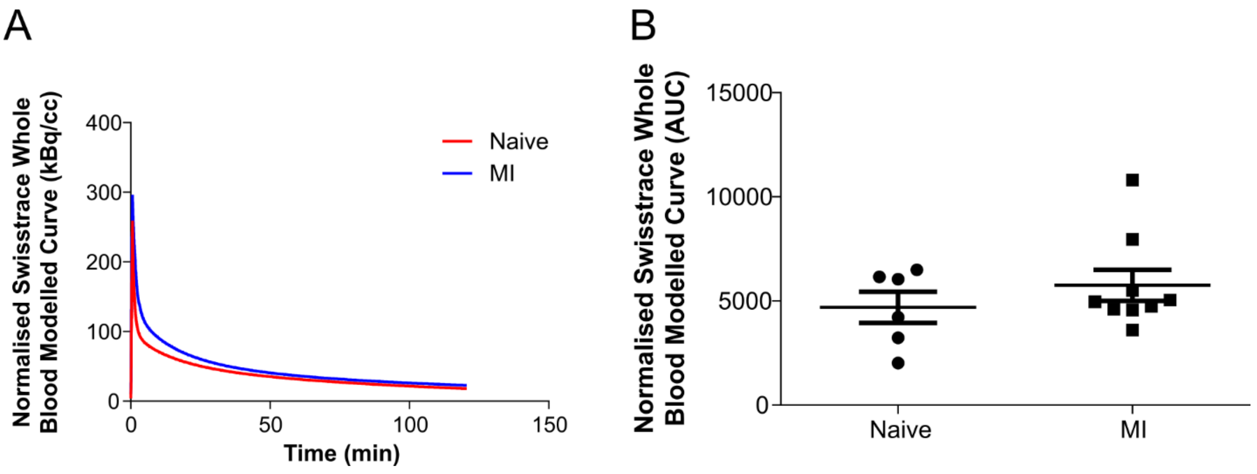

Supplemental Figure 11. Invasive input function measurements in naive and MI rats during ^{18}F-LW223 PET. A) Average time activity curve of whole blood modelled and normalized to injection dose. **B)** Area under the curve for each individual blood curve normalized to injection dose. Naive *n* = 6, MI *n* = 9, mean ±SEM.

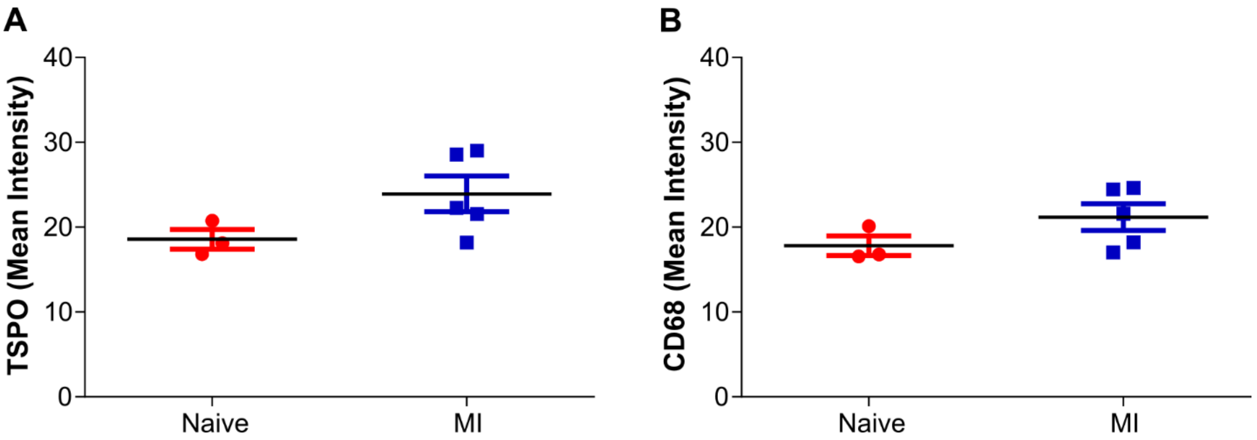

Supplemental Figure 12. *Ex-vivo* immunofluorescent staining within the posterior left ventricle. A) TSPO expression sampled within the posterior wall of the left ventricle in naive and MI hearts. **B)** CD68 expression sampled within the posterior wall of the left ventricle in naive and MI hearts. Results represent the mean±S.E.M., naive n = 3, MI n = 5.

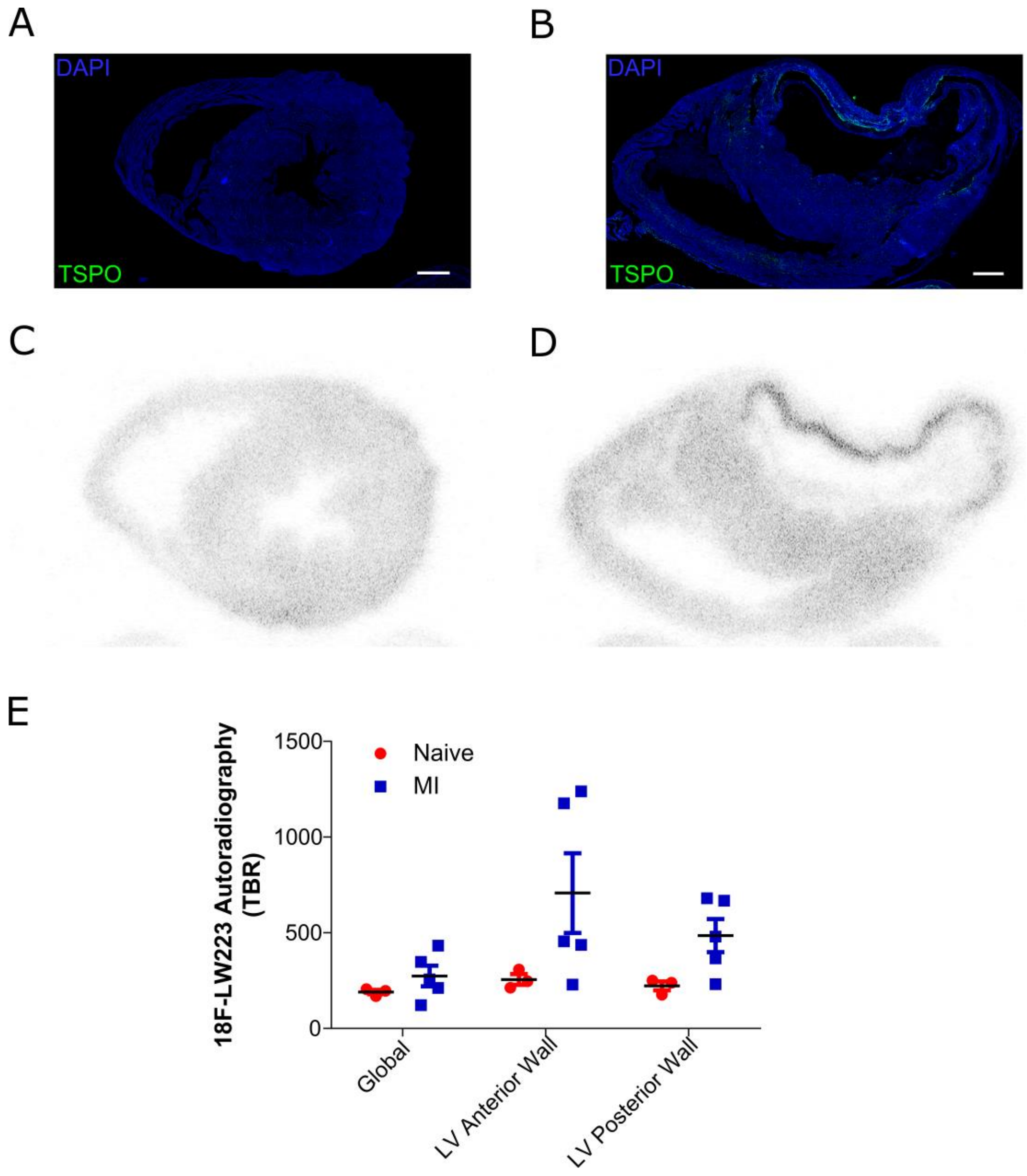

Supplemental Figure 13. ^{18}F-LW223 Autoradiography. A) Example TSPO immunofluorescent staining in naive and **B)** MI hearts. **C)** Example autoradiography image of the same naive and **D**) MI heart. **E)** Quantification of the autoradiography in the global heart and left ventricle (LV) anterior wall. Results represent the mean±S.E.M., naive *n* = 3, MI *n* = 5.

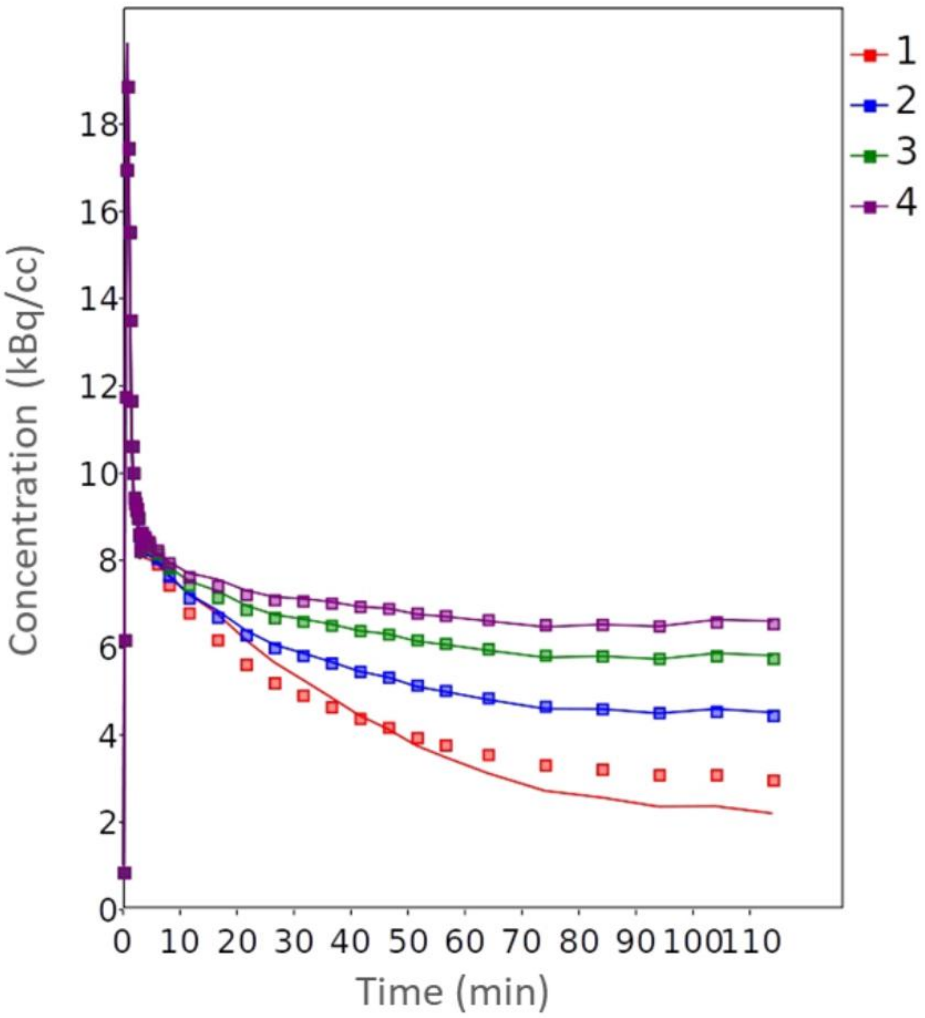

Supplemental Figure 14. Simulation on the effect of changes in K_1 on *BP_{ND}*. Example of simulated curves with lowest K_1=0.01 mL/mim/cc and 4 different BP_{ND} values (1= BP_{ND} of 4; 2= BP_{ND} of 10; 3= BP_{ND} of 16; and 4= BP_{ND} of 20). All simulated curves are noise free.

Curve	K_1 simulated	k_2 simulated	k_3 simulated	k_4 simulated	BP_{ND} simulated
**1**	0.9	0.3	0.02	0.005	4
**2**	0.9	0.3	0.05	0.005	10
**3**	0.9	0.3	0.08	0.005	16
**4**	0.9	0.3	0.1	0.005	20
**5**	0.7	0.3	0.02	0.005	4
**6**	0.7	0.3	0.05	0.005	10
**7**	0.7	0.3	0.08	0.005	16
**8**	0.7	0.3	0.1	0.005	20
**9**	0.5	0.3	0.02	0.005	4
**10**	0.5	0.3	0.05	0.005	10
**11**	0.5	0.3	0.08	0.005	16
**12**	0.5	0.3	0.1	0.005	20
**13**	0.3	0.3	0.02	0.005	4
**14**	0.3	0.3	0.05	0.005	10
**15**	0.3	0.3	0.08	0.005	16
**16**	0.3	0.3	0.1	0.005	20
**17**	0.1	0.3	0.02	0.005	4
**18**	0.1	0.3	0.05	0.005	10
**19**	0.1	0.3	0.08	0.005	16
**20**	0.1	0.3	0.1	0.005	20
**21**	0.01	0.3	0.02	0.005	4
**22**	0.01	0.3	0.05	0.005	10
**23**	0.01	0.3	0.08	0.005	16
**24**	0.01	0.3	0.1	0.005	20

Supplemental Table 5. Kinetic parameters used in simulations. The blood volume (vB) was kept at 5% or 0.05 for all simulations. These parameters were based on the results of the modelling presented in the paper.

Curve	K_1 estimated	K_1 %SD	k_2 estimated	k_3 estimated	k_4 estimated	BP_{ND} estimated	BP_{ND} %SD	BP_{ND} simulated – BP_{ND} estimated	BP_{ND} % difference	BP_{TC} estimated	Weighted sum of squares	2TCM AIC
**1**	0.844	0.367	0.276	0.019	0.005	4.106	4.689	-0.106	-2.648	4.865	58.295	24.853
**2**	0.841	0.416	0.271	0.048	0.005	9.839	2.044	0.161	1.611	11.703	55.936	23.242
**3**	0.838	0.442	0.266	0.077	0.005	15.422	1.366	0.578	3.613	18.410	55.344	22.827
**4**	0.836	0.455	0.263	0.096	0.005	19.004	1.185	0.996	4.980	22.737	55.372	22.847
**5**	0.656	0.396	0.275	0.019	0.005	4.127	4.397	-0.127	-3.183	6.289	36.972	7.094
**6**	0.654	0.428	0.270	0.048	0.005	9.844	1.931	0.156	1.560	15.061	35.091	5.058
**7**	0.651	0.415	0.265	0.077	0.005	15.374	1.413	0.626	3.913	23.612	34.263	4.126
**8**	0.650	0.462	0.262	0.096	0.005	18.957	1.212	1.043	5.215	29.187	34.019	3.848
**9**	0.467	0.429	0.274	0.019	0.005	4.131	5.080	-0.131	-3.270	8.851	20.523	-15.860
**10**	0.465	0.387	0.269	0.048	0.005	9.832	1.802	0.168	1.680	21.153	19.270	-18.320
**11**	0.463	0.497	0.263	0.077	0.005	15.347	1.490	0.653	4.081	33.154	18.721	-19.450
**12**	0.462	0.282	0.260	0.096	0.005	18.900	1.313	1.100	5.500	40.936	18.504	-19.900
**13**	0.279	0.467	0.271	0.019	0.005	4.163	6.155	-0.163	-4.062	14.946	9.359	-46.490
**14**	0.277	0.469	0.266	0.048	0.005	9.824	2.093	0.176	1.758	35.441	8.706	-49.310
**15**	0.276	0.507	0.260	0.076	0.005	15.284	1.480	0.716	4.475	55.397	8.418	-50.620
**16**	0.275	0.472	0.256	0.095	0.005	18.802	1.310	1.198	5.990	68.321	8.280	-51.260
**17**	0.090	0.664	0.260	0.018	0.004	4.354	12.591	-0.354	-8.838	48.158	3.830	-81.330
**18**	0.090	0.891	0.252	0.046	0.005	9.818	4.715	0.182	1.824	109.327	3.573	-84.040
**19**	0.089	0.897	0.244	0.074	0.005	15.011	3.165	0.989	6.181	168.285	3.281	-87.370
**20**	0.089	0.901	0.238	0.092	0.005	18.292	2.703	1.708	8.540	205.991	3.183	-88.550
**21**	0.007	7.955	0.161	2.000	3008.983	50.000	43.722	-46.000	-1150.000	7616.842	7.101	-57.250
**22**	0.006	6.791	0.137	0.033	0.003	10.833	164.239	-0.833	-8.330	1682.012	3.120	-89.330
**23**	0.006	6.191	0.116	0.050	0.004	12.443	113.252	3.557	22.231	1961.937	3.070	-89.960
**24**	0.006	6.417	0.103	0.059	0.004	13.208	91.255	6.792	33.960	2100.108	3.043	-90.300

Supplemental Table 6. Estimated kinetic parameters from simulations. Curve numbers related to curve simulated kinetic parameters in Supplemental Table 5. K_1 is relatively well estimated, however the BP_{ND} becomes unstable at low levels of K_1.

Time (min)	Activity (kBq/cc)
0.08	20.06
0.25	127.34
0.42	237.26
0.58	356.85
0.75	387.21
0.92	345.15
1.08	298.41
1.25	253.76
1.42	211.44
1.58	187.91
1.75	175.63
1.92	160.92
2.08	158.90
2.25	153.93
2.42	154.93
2.58	149.61
2.75	140.65
2.92	131.96
3.25	133.83
3.75	129.21
4.50	125.97
6.00	120.01
8.00	110.94
11.50	99.15
16.50	89.07
21.50	78.29
26.50	70.74
31.50	66.03
36.50	61.30
41.50	56.47

46.50	53.55
51.50	49.17
56.50	46.83
64.00	42.98
74.00	37.98
84.00	36.45
94.00	33.08
104.00	33.56
114.00	30.45

Supplemental Table 7. Input function data used for simulation work.

Time (min)	Parent fraction
2	0.95
5	0.82
10	0.81
20	0.81
30	0.77
60	0.54
120	0.69

Supplemental Table 8. Radiometabolite data used for simulation work.

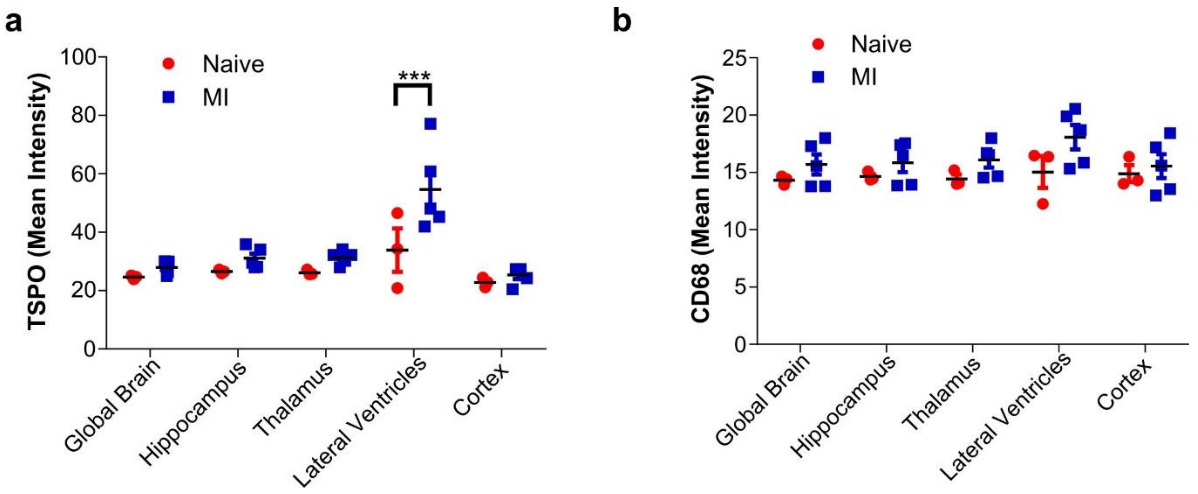

Supplemental Figure 15. Quantification of TSPO and CD68 immunofluorescent staining. A) TSPO signal within the brain and selected sub-regions of naive and MI rats. **B)** CD68 signal within the brain and selected sub-regions of naive and MI rats. Results represent the mean±S.E.M., naive *n* = 3, MI *n* = 5.
